# Perturbation of placental protein glycosylation by endoplasmic reticulum stress promotes maladaptation of maternal hepatic glucose metabolism

**DOI:** 10.1016/j.isci.2022.105911

**Published:** 2022-12-30

**Authors:** Hong Wa Yung, Xiaohui Zhao, Luke Glover, Charlotte Burrin, Poh-Choo Pang, Carolyn J.P. Jones, Carolyn Gill, Kate Duhig, Matts Olovsson, Lucy C. Chappell, Stuart M. Haslam, Anne Dell, Graham J. Burton, D. Stephen Charnock-Jones

**Affiliations:** 1 Centre for Trophoblast Research, University of Cambridge, Cambridge CB2 3EG, UK; 2 Department of Life Sciences, Imperial College London, London, UK; 3 Maternal and Fetal Health Centre, University of Manchester, Manchester Academic Health Sciences Centre, Manchester, UK; 4 Department of Women and Children’s Health, King’s College London, London, UK; 5 Department of Women’s and Children’s Health, Uppsala University, Uppsala, Sweden; 6 Department of Obstetrics and Gynaecology, University of Cambridge, Cambridge CB2 0SW, UK

**Keywords:** Pregnancy, Biological sciences, Human metabolism

## Abstract

Placental hormones orchestrate maternal metabolic adaptations to support pregnancy. We hypothesized that placental ER stress, which characterizes early-onset pre-eclampsia (ePE), compromises glycosylation, reducing hormone bioactivity and these maladaptations predispose the mother to metabolic disease in later life. We demonstrate ER stress reduces the complexity and sialylation of trophoblast protein N-glycosylation, while aberrant glycosylation of vascular endothelial growth factor reduced its bioactivity. ER stress alters the expression of 66 of the 146 genes annotated with “protein glycosylation” and reduces the expression of sialyltransferases. Using mouse placental explants, we show ER stress promotes the secretion of mis-glycosylated glycoproteins. Pregnant mice carrying placentas with junctional zone-specific ER stress have reduced blood glucose, anomalous hepatic glucose metabolism, increased cellular stress and elevated DNA methyltransferase 3A. Using pregnancy-specific glycoproteins as a readout, we also demonstrate aberrant glycosylation of placental proteins in women with ePE, thus providing a mechanistic link between ePE and subsequent maternal metabolic disorders.

## Highlights

• ER stress promotes the secretion of mis-glycosylated proteins
• Mis-glycosylated protein looses normal biological activity
• Placenta-specific ER stress causes mal-adaptation of maternal glucose metabolism
• The placenta in early-onset pre-eclampsia releases mis-glycosylated proteins

## Graphical abstract

**Figure:**
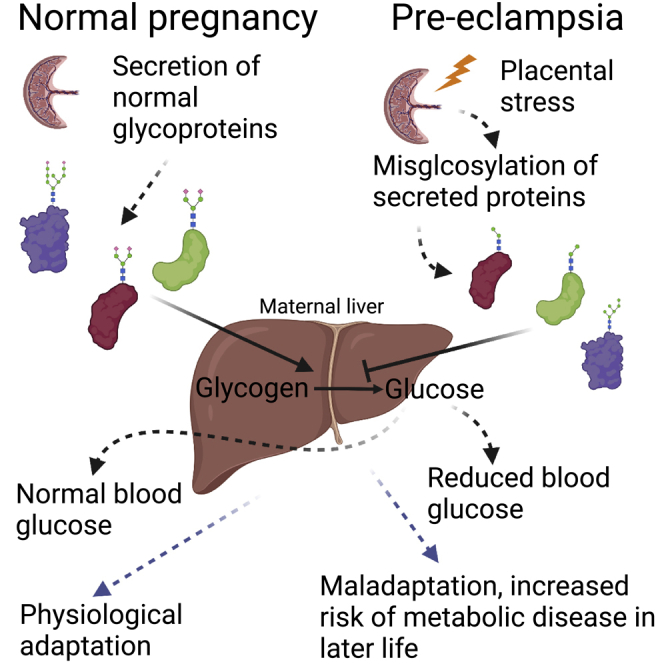

## Introduction

Successful pregnancy requires fine-tuned sharing of resources between mother and fetus to support proper growth of the fetal-placental unit without compromising maternal health and survival.^1^^,^^2^ Extensive maternal adaptations ensure an adequate supply of nutrients and oxygen to the growing fetus. There are also fetal adaptations and all these adaptations are sometimes described as maternal-fetal conflict. They include increased peripheral insulin resistance to mobilize glucose, amino acids, and lipids for transfer, and cardiovascular changes.^3^^,^^4^ These adaptations are largely orchestrated by placental hormones. Increasing evidence indicates that pregnancies complicated by placental dysfunction, including fetal growth restriction (FGR) and/or pre-eclampsia (PE), adversely influence the woman’s long-term health, increasing the risk of developing cardiovascular disease, type II diabetes, and obesity in later life 3- to 10-fold.^5^^,^^6^^,^^7^^,^^8^

The placenta plays a central role in maternal-fetal resource allocation through monitoring of maternal nutrient availability.^2^ Maternal energy reserves are laid down during early to mid-gestation and mobilised to support rapid fetal growth during the second half.^2^ Increased maternal appetite and leptin resistance initially promote lipid accumulation,^2^ while developing maternal peripheral insulin resistance later increases hepatic gluconeogenesis, reduces glucose uptake in maternal skeletal muscle and adipose tissue, and increases lipolysis, releasing glucose and lipids for transfer to the fetus.^9^^,^^10^ The liver plays an essential role in glucose homeostasis by regulating various glucose metabolic pathways.^11^ Maternal hepatic physiology and function adjust accordingly as pregnancy progress, including increased cell proliferation and mass,^12^ and elevated basal endogenous glucose production by 30%.^13^ However, in pre-eclamptic pregnancies, these changes are either impaired or exacerbated.^14^^,^^15^

A number of placentally derived factors (including placental growth hormone variant, placental lactogen, adiponectin, and insulin-like growth factor-binding proteins) contribute to the maternal metabolic changes and concentrations of these factors increase throughout pregnancy.^2^^,^^16^ The majority are synthesized in the rough ER where folding and glycosylation of nascent polypeptides are also affected. Protein glycosylation links oligosaccharides side chains or glycans to the nitrogen (*N*-glycans) and oxygen (*O*-glycans) of asparagine and serine/threonine respectively. N-linked glycosylation is initiated in the ER and completed in the Golgi complex. A family of glycosyltransferases and glycosidases regulates the formation of glycans in a stepwise fashion, influenced by substrate availability, gene transcription, enzyme activity, and enzyme location within the organelles.^17^ The process is completed by sialic acid end-capping, protecting glycoproteins from systemic clearance.^18^ Therefore, correct glycosylation is essential for circulating protein half-life, receptor affinity and antigenicity.^18^^,^^19^^,^^20^ Cells and organs suffering from ER stress secrete aberrantly glycosylated proteins^21^^,^^22^ which been closely linked to various diseases.^17^

We have previously shown the presence of ER stress in placentas from pregnancies complicated by early-onset pre-eclampsia (ePE), FGR, and gestational diabetes.^23^^,^^24^ Consequently, we hypothesize that mis-glycosylation may compromise the activity and bioavailability of placental factors, thereby adversely affecting maternal physiological adaptations, placing stress on organ systems and potentially increasing susceptibility to the future development of metabolic diseases. To address this hypothesis, we investigated whether placental ER stress altered the function of placentally derived factors and perturbed maternal physiological adaptation. We first used an *in vitro* cell model and a novel *ex vivo* junctional zone (Jz) placental explant model to determine whether ER stress can indeed alter the glycosylation of placentally derived glycoproteins. We used transcriptome analysis and a functional bioassay to investigate the molecular mechanisms and consequences of mis-glycosylation. We subsequently generated a transgenic mouse model with placental junctional zone-specific deletion of *Perk (Jz-Perk*^*−/−*^*)*. We examined whether chronic ER stress generated in the junctional zone (the region of the placenta responsible for endocrine activity) perturbs maternal physiology, focusing on maternal hepatic glucose metabolism. Finally, we sought evidence for aberrantly glycosylated proteins in samples from human pregnancies complicated by early-onset pre-eclampsia, focusing on pregnancy-specific glycoproteins (PSGs) that are secreted largely by the placenta, although low levels of the PSGs are also expressed in the gastrointestinal tract but at levels several orders of magnitude lower.^25^

## Results

### Endoplasmic reticulum stress alters glycosylation pattern of secretory proteins

**Figure 1:**
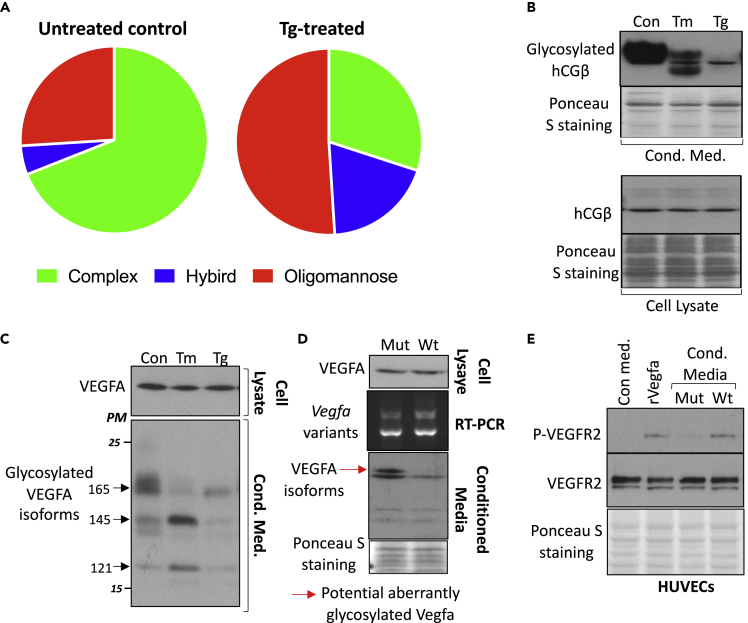
ER stress alters protein glycosylation and function BeWo cells, grown in serum-free medium were treated with ER stress inducers, thapsigargin (Tg) (500 nM) or tunicamycin (Tm) (1 μg/mL) for 24 h. Conditioned media were harvested and cell lysates prepared for glycomic or Western blotting analysis. (A) Glycomic analysis of the trophoblastic BeWo cell secretome. N-glyans were released from the secreted protein and analyzed by MALDI-TOF MS. The pie charts summarize the distribution of three glycan subtypes: oligomannose, hybrid, and complex structure glycans in secreted glycoproteins from untreated control and Tg-treated BeWo cells respectively. The pie charts were plotted from the average value of 2 independent experiments. (B) ER stress reduces the overall glycosylation of secreted hCG. Western blotting for hCG β chain in conditioned media and cell lysates prepared from BeWo cells treated with Tm or Tg. (C-E) ER stress-induced aberrant glycosylation of VEGFA reduces its bioactivity. (C) Cell lysates and conditioned media were collected from BeWo cells after Tg or Tm treatment for Western blot analysis of level of cellular and secreted VEGFA. (D-E) Alteration of glycosylation and loss of bioactivity in secreted VEGFA by *Eif2s1*^tm1RjK^ mutant MEFs. (D) *Eif2s1*^*tm1Rjk*^ homozygous mutant MEFs showed no change in *Vegfa* transcripts (detected by RT-PCR) and cellular protein but an altered band pattern of secreted VEGFA (red arrow). Western blot was used to visualize cellular VEGFA and secreted VEGFA isoforms in cell lysates and conditioned media. (E) VEGFA secreted by *Eif2s1*^*tm1Rjk*^ homozygous mutant MEFs fails to activate KDR/VEGFR2 in endothelial cells. Human umbilical vascular endothelial cells (HUVECs) were pre-treated with serum-free medium for 6 h before challenge with conditioned media, 25 ng/mL recombinant Vega, or control medium for 10 min. Anti-P-VEGFR2 antibody was used to detect the activation of VEGFR2. Ponceau S staining showed equal protein loading among samples. wt, wild type fibroblasts; Mut, *Eif2s1*^tm1RjK^ mutant fibroblasts; P-VEGFR2, phosphorylated VEGFR2; PM, protein marker.

To investigate whether ER stress underpins the secretion of mis-glycosylated placental glycoproteins, we treated trophoblast-like BeWo cells in the serum-free medium with thapsigargin (Tg), an ER stress inducer and sarco/ER Ca^2+^-ATPase inhibitor, following by glycomic analysis of conditioned media. Primary trophoblasts were not used because their isolation and culture induce substantial ER stress that would likely confound the data.^26^ The N-glycans in the conditioned medium were released from the glycoproteins and analyzed by Matrix-Assisted Laser Desorption/Ionization-Time of Flight (MALDI-TOF) mass spectrometry. N-linked glycan structures can be classified into three major subtypes based on their degree of processing: oligomannose, hybrid, and complex. As shown in Figure S1, both control and Tg-treated samples contained a diverse mixture of oligomannose, hybrid, and complex N-glycans. In untreated conditioned medium, the proportions of glycans with oligomannose, hybrid, and complex structures were 26%, 5%, and 69% respectively. In comparison, under ER stress conditions, these ratios shifted to 51%, 19%, and 30% respectively (Figure 1A). In addition, ER stress reduced the sialylation of complex N-glycans indicated by a decrease in the relative abundance of fully sialylated species (*m*/*z* 3776) in comparison to partially and un-sialylated species (*m*/*z* 2693, 3054, 3415) (blue peaks inside the blue shaded area in Figure S1). These results are consistent with the study by Wong et al.^22^ We did not detect any glycan structures containing *N*-Glycolylneuraminic acid (NeuGc), which is only found in non-human mammals. As humans are unable to synthesize this (due to a mutation in CMP-N-acetylneuraminic acid hydroxylase) this suggests that possible contamination with FBS is low.

While mass spectrometry gives an overview of a shift in the overall composition of the glycome within a sample, it does not allow the direct comparison of the absolute abundance of any specific glycan between two samples. We, therefore, sought to use a placental-specific glycoprotein as an example to investigate the overall change of degree of glycosylation in the protein under ER stress. Human chorionic gonadotrophin (hCG), one of the principal secretions of trophoblast, contains two heavily N-glycosylated subunits α and β.^27^ We used Western blotting analysis because the degree of glycosylation and/or the complexity of glycan structures can be detected by the pattern, intensity and molecular weights of hCG band(s). Following treatment with Tg or another ER stress inducer, tunicamycin (Tm) (which inhibits N-glycoslyation and therefore can be used as a positive control) glycosylation of the secreted hCGβ chain was markedly decreased (Figure 1B). The effect was especially prominent following Tg treatment when little glycosylated hCG was observed. This suggests that ER stress not only impairs the complexity of glycans being synthesized but also greatly reduces the overall level of protein glycosylation.

The changes in complexity and amount of glycosylation may perturb the function and activity of secreted glycoproteins. To test this, we selected vascular endothelial growth factor A (VEGFA) as an exemplar in preference to hCG to assess the effects of ER stress on protein activity. Bioassays for hCG typically use fresh testicular cells collected from mice or rats. The complexity and the use of live animals make this bioassay unattractive. In contrast, VEGFA rapidly induces the phosphorylation of VEGF receptor 2 (VEGFR2) and thus the activity of VEGFA can be evaluated directly using readily available human umbilical vein endothelial cells (HUVECs). This cell-based bioassay is rapid and highly specific for VEGFA. The treatment of BeWo cells with Tg and Tm altered the glycosylation profiles of different isoforms of VEGFA in the conditioned medium (CM) (Figure 1C, black arrows) confirming that ER stress alters the pattern and amount of glycosylation of secreted glycoproteins. However, the conditioned medium could not be used directly in an assay as residual Tg and Tm in the conditioned medium confound this experiment. Since VEGFA also expressed by human trophoblast^28^ and by mouse embryonic fibroblasts (MEFs) a genetic model of ER stress can be used which avoids interference from any residual ER stress inducer present. Human VEGFA and mouse VEGFA are highly homologous (83.4% protein sequence identity) and both contain a single glycosylation site (UniportKB-Q00731). We, therefore, used a genetic model of ER stress, *Eif2s1*^tm1RjK^ in which serine 51 is replaced by alanine in eIF2α resulting in continuous ER stress in homozygous mutant mouse embryonic fibroblasts (MEFs).^29^^,^^30^ It is worth noting that Tg leads protein misfolding through the disruption of ER Ca^2+^ homeostasis while in the *Eif2s1*^*tm1RjK*^ mutant cells, the high protein translation rate overwhelms the protein folding machinery resulting in protein misfolding. Although their precise effects on the protein glycosylation profile are likely to be different, the aberrant glycosylation in both conditions is likely to have a similar consequence on the protein function/activity. We first determined levels of *Vegfa* transcripts and cellular protein by RT-PCR and Western blotting. There was no difference between wild-type (wt) and *Eif2s1*^tm1RjK^ mutant (Mut) MEFs (Figure 1D). Despite no change in cellular VEGFA protein level, we observed an increase in the highest molecular weight VEGFA and an extra band of VEGFA in CM from the mutant MEFs (Figure 1D, red arrow). To evaluate the biological activity of the VEGFA in the CM we briefly exposed human umbilical vascular endothelial cells (HUVECs) to CM harvested from either wt or Mut MEFs. As expected, both wt CM and recombinant VEGFA (rVEGFA) induced rapid phosphorylation of VEGFR2. However, CM from the Mut MEFs failed to do so, indicating the loss of VEGFA bioactivity (Figure 1E). We have previously reported that conditioned medium from *Eif2s1*^tm1RjK^ mutant MEFs failed to maintain trophoblast stem cells in a pluripotent state,^30^ consistent with loss of functional activity of other secreted growth factors.

### Endoplasmic reticulum stress modulates the expression of genes involved in glycan biosynthesis

We next investigated the mechanisms by which ER stress influences protein glycosylation using RNA-Seq. We generated RNA-Seq datasets from BeWo cells treated with and without Tg (5 independent replicate pairs). Read numbers and alignment rates are given in Table S1. Principal Component Analysis (PCA) showed two distinct groups of samples with separation dependent on Tg treatment (Figure S2A). Differential expression analysis using DESeq2 showed there were 1712 and 1482 transcripts increased and decreased respectively following Tg treatment (P_adj_ < 0.05 and absolute fold-change ≥2, Table S2; differentially expressed genes, DEGs).

**Figure 2:**
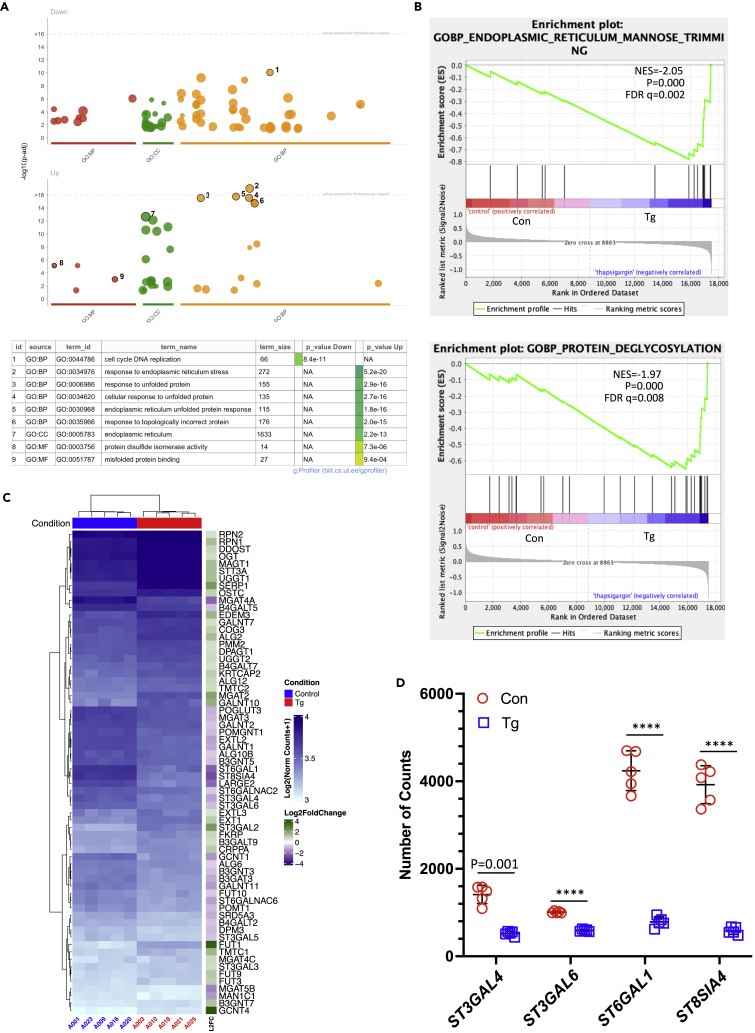
Genomic analysis in BeWo cells after Tg treatment RNA-Seq was used to analyze transcript changes induced by Tg in BeWo cells. (A) g.Profiler analysis with GO_BP revealed biological processes affected by thapsigargin treatment. (B) GSEA with GO_BP showed enrichment of transcripts required for Mannose Trimming, a key process in glycosylation and Protein Deglycosylation. (C) Heatmap of 66 differentially expressed genes (FC > 1.5, P_adj_ < 0.05) induced by Tg treatment involved in protein glycosylation from gene ontology (GO: 0006486). (D) Sialyltransferases involved in N-glycan sialylation are down-regulated in Tg-treated BeWo cells. The number of normalized counts from RNA-Seq is plotted. Data are mean ± SD, n = 5. Paired t-test. ∗∗∗∗ indicates p < 0.0001. NES, normalized enrichment score; Con, control; Tg, Thapsigargin.

To investigate how Tg affects biological processes in an unbiased manner we used gprofiler2 to identify over-represented GO terms related to up- and down-regulated genes. Terms associated with the cell cycle and DNA replication were over-represented in the down-regulated genes. For example, “cell cycle DNA replication,” (GO:0044786, P_adj_ = 8.4 × 10^−11^). Among the up-regulated genes, many terms related to ER stress were enriched, for example “response to ER stress” GO:0034976, P_adj_ = 5.2 × 10^−20^ (Figure 2A). Many of the most differentially regulated genes are annotated with these or related terms as highlighted in the heatmap of the top 100 DEGs ordered by P_adj_ (from smallest to largest) (Figure S2B). Furthermore, Gene Set Enrichment Analysis (GSEA) supported this finding with GOBP_ER_Unfolded Protein Response being top of the GSEA report (NES = −2.68, p = 0.000 and FDR = 0.000, Figure S3A; Tables S3 and S4).

Having observed changes in the glycan profile of the conditioned media (Figures 1A and 1B; S1), we noted Tg treatment also changed the transcript levels of genes involved in protein glycosylation. In the GSE analysis GOBP_ER_Mannose_Trimming (NES = −2.05, p = 0.000 and FDR q = 0.002) and GOBP_Protein_Deglycosylation (NES = −1.97, p = 0.000 and FDR q = 0.008) were ranked 17^th^ and 21^st^ respectively indicating the alteration of protein glycosylation upon ER stress (Figure 2B). These gene sets are listed as heatmaps and tables (Figure S3B; Tables S5 and S6).

146 genes associated with the GO:BP term “Protein glycosylation,” GO:0006486, are present in the dataset and 66 were identified as differentially regulated by Tg treatment (P_adj_ < 0.05 and absolute fold-change ≥1.5, Table S7). This is very significant over-representation (Fisher exact test, two-sided, p = 2.2 × 10^−16^ and the genes are shown in a heatmap (Figure 2C). Sialyltransferases involved in N-linked glycosylation include ST3GAL4 & ST3GAL6 for α2,3 linkages; ST6GAL1 and ST6GAL2 for α2,6 linkages and ST8SIA2 and ST8SIA4 for α2,8 linkages.^31^ The mRNAs encoding *ST3GAL4*, *ST3GAL6*, *ST6GAL1*, and *ST8SIA4* were also reduced in Tg-treated BeWo cells (Figure 2D) while *ST6GAL2* and *ST8SIA2* mRNAs had very low (<4) and no counts respectively. This would explain the loss of sialylation observed in the glycomic analysis above (Figure S1). Changes in genes involved in N-glycosylation are illustrated in the KEGG pathway for N-glycan biosynthesis (KEGG: 00510, Figure S4).

### Endoplasmic reticulum stress induces placental tissues to release aberrantly glycosylated proteins

To determine whether these cell culture-based findings occur in the placenta *in vivo*, we established a novel mouse placental junctional zone explant culture *ex vivo* model. The mouse placenta has two distinctive regions, the junctional zone (Jz) for endocrine activity and labyrinthine zone (Lz) for nutrient and gaseous exchange. The Jz is an active secretory tissue, confirmed by immunochemical staining of the ER chaperone GRP78 which is almost exclusively localized to the Jz (Figure S5A). We dissected the placenta into decidua, Jz and Lz (Figure S5B) and estimated the purity by RT-qPCR using Jz and Lz marker genes (*Tpbpa* and *Gcm1* respectively). Dissected Jz was contaminated with Lz (11.9% ± 4.4%, mean ± SD) and dissected Lz contaminated with Jz (1.2% ± 0.4%, mean ± SD, n = 4, Figure S5C). We assessed the viability of the explants after 48 h in culture using the MTT assay which confirmed the presence of functional mitochondria (Figure S5D). Furthermore, phosphorylated AKT (a cell survival and proliferation kinase) was increased whereas levels of ER stress markers (P-EIF2α and ATF4), and phosphorylation of stress kinases JNKs and energy sensing kinase AMPKα were lower than observed at t = 0 (Figure S5E).

**Figure 3:**
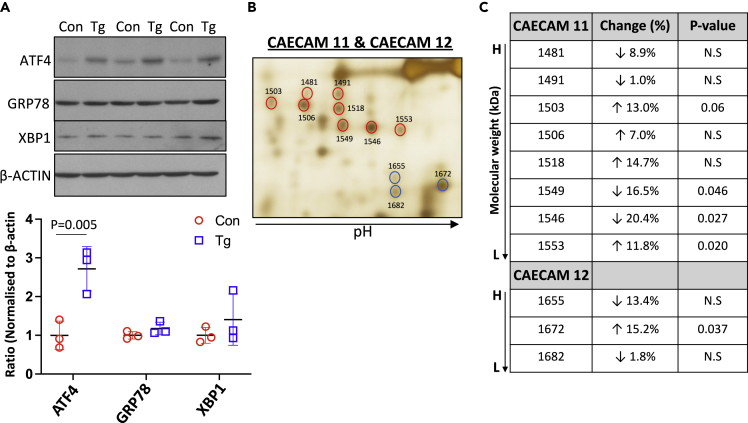
ER stress promotes mis-glycosylation of secretory proteins in murine Jz explant culture Jz explants were treated with Tg (100 nM) for 48 h. (A) Tg treatment induces low-grade ER stress in Jz explants. After treatment, Jz homogenate was analyzed by Western blotting for the ER stress markers ATF4, GRP78, and XBP1; β-ACTIN was used as loading control. Band intensities were quantified and data normalized to the mean value of untreated control. Data are presented as mean ± SD, n = 3. Paired t-test. (B) DIGE reveals changes in glycosylation after Jz culture with Tg treatment. Circled spots were excised and identified by mass spectrometry as spongiotrophoblast-specific CEACAM11 (red circles) and CEACAM12 (blue circles). (C) Tg treatment induces glycosylation changes in CEACAM11 and CEACAM12. The level of each spot was normalized to the mean expression level of the protein. A reduction in molecular weight indicates loss of glycan complexity while increased pI of the spot suggests potential loss of sialic acid group(s).

Treatment with Tg (100 nM) induced mild ER stress in Jz explants, causing a 2.6-fold (p = 0.005) increase in ATF4, while GRP78 and XBP1 remained unchanged after 48 h, indicating the activation of low-grade ER stress (Figure 3A). Both the study from Wong et al. 22 and our study (Figures 1A and S1) have shown that Tg treatment alters the glycosylation pattern of secretory proteins - increasing glycans with oligomannose while decreasing glycans with sialyation on specific N-glycan epitopes. This would lead to a change of molecular weight and isoelectric focusing point (pI) of the mis-glycosylated glycoprotein. Therefore, we analyzed proteins in conditioned media from both untreated and Tg-treated Jz explants using two-dimension fluorescence Difference Gel Electrophoresis (DIGE). Nineteen of the differentially expressed spots (p < 0.05, Figure S5F) were selected, excised, digested with trypsin, and identified by LC-MS/MS proteomic analysis. Eighteen of the spots were clustered closely together, indicating likely changes in glycan structure. The identities of these spots are listed in Figure S5G. Indeed, among these were two clusters containing 8 and 3 spots which we identified as two members of the Carcinoembryonic Antigen-Related Cell Adhesion Molecules (CEACAM) family, (CEACAM11 and CEACAM12). The murine CEACAM family is closely related to the human pregnancy-specific glycoprotein (PSG) family.^32^ Both CEACAM11 and CEACAM12 contain 4 glycosylation sites and are synthesized and secreted by the mouse placenta.^32^ We detected Tg-induced changes in molecular weight and pl (Figure 3B, red and blue circles) with changes in the spot intensity ratios. For example, for CEACAM11, spots 1503 and 1553 increased by 13% (p = 0.06) and 11.8% (p = 0.02) while spots 1549 and 1546 decreased by 16.5% (p = 0.046) and 20.4% (p = 0.027) respectively (Figure 3C). Spots with reduced molecular weight reflect a reduction in glycan complexity while spots with increased pI suggested potential loss of sialic acid end-cap(s). For instance, the Tg-mediated decrease in CEACAM11 spots 1549 and 1546 was associated with increased protein level of spot 1553, which has a slightly reduced molecular weight and a higher pI, suggesting potential loss of sialic acid(s). These examples further support the hypothesis that ER stress in placental tissue can cause the secretion of aberrantly glycosylated proteins. Therefore, we next investigated whether ER stress-mediated perturbation of placental endocrine function affects maternal physiological adaptations to pregnancy.

### Jz-specific endoplasmic reticulum stress reduces placental efficiency and increases embryonic lethality

We generated a placental-specific ER-stress mouse model in which *Perk* (PRKR-like ER kinase) that encodes one of the three sensors in the ER unfolded protein response (UPR) signaling pathways is selectively ablated in the junctional zone (*Jz-Perk*^*−/−*^) using Cre recombinase driven by the *Tpbpa* promotor. This is active in ectoplacental cone (EPC) cells starting between embryonic days E7.5 and 8.5, and later in the spongiotrophoblast layer of the mature placenta. EPC-derived cells include spiral artery trophoblast giant cells (TGCs), canal TGCs, and trophoblast glycogen cells as well as the more numerous spongiotrophoblast cells.^33^ However, it is the spongiotrophoblast cells that have high secretory activity as demonstrated by strong staining for the ER chaperone GRP78. The trophoblast glycogen cells in the Jz do not stain for this marker (Figure S5A, inset red arrows). The *Tpbpa-cre* allele is inherited from the sire so the only tissues that could express cre are embryonic, fetal, or placental and *in situ* hybridization to detect *Tpbpa* mRNA in the mouse placenta at E14 shows signal exclusively in the Jz.^34^ Activation of the PERK pathway maintains ER function and is part of a homeostatic response, and loss of *Perk* leads to hypersensitivity to ER stress.^35^ The degree of placental ER stress under normal physiological conditions is minimal and there were no differences in litter size, embryonic loss, placental weight, fetal weight and placental efficiency between *Perk*^*fl/fl*^ (control) or *Jz-Perk*^*−/−*^ litters (Figures S6A and S6B). There is a close relationship between hypoxia and ER stress^36^ and we previously demonstrated that hypoxia specifically activates the PERK arm of the ER-UPR pathway in both *in vitro* and *in vivo* models.^37^^,^^38^ Therefore, to cause ER stress we housed all pregnant females in 13% O_2_ from embryonic day 0.5 (E0.5) to E18.5.

**Figure 4:**
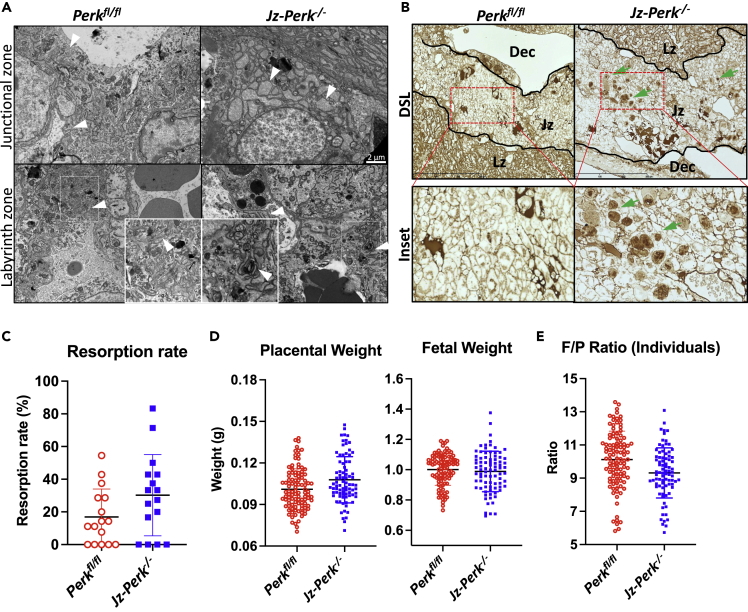
ER stress in the *Jz-Perk*^*−/−*^ placenta and its consequence on pregnancy outcome Females carrying *Perk*^*fl/fl*^ or *Jz-Perk*^*−/−*^ litters were housed under reduced oxygen (13% O_2_) from E0.5 and all parameters were measured and tissues were collected at E18.5. (A) Electron micrographs exclusively show the dilatation of ER cisternae in Jz of *Jz-Perk*^*−/−*^ placenta (arrowheads), indicating loss of ER homeostasis. (B) Lectin staining with *Datura stramonium* lectin (DSL) with preferential binding to GlcNAc reveals protein aggregates in Jz of *Jz-Perk*^*−/−*^ placenta (examples highlighted with green arrows), indicating potential protein mis-glycosylation. (C) Resorption rate in females carrying *Jz-Perk*^*−/−*^ placentas. Data are presented as median with 95% Cl, *Jz-Perk*^*−/−*^ and *Perk*^*fl/fl*^ = 16 litters each, Mann Whitney test. (D) A trend to increased placental weight with no change in fetal weight in *Jz-Perk*^*−/−*^ animals. (E) No change in placental efficiency in litters with *Jz-Perk*^*−/−*^ placentas. Data are presented as mean ± SD, *Jz-Perk*^*−/−*^ = 82 fetuses; *Perk*^*fl/fl*^ = 104 fetuses. D & E) Nested t-test.

Housing under reduced oxygen resulted in increased phosphorylation of EIF2α in Jz of *Perk*^*fl/fl*^ placentas, but this was reduced in the *Jz-Perk*^*−/−*^ placentas while there was no difference in GRP78 and spliced variant of XBP1 protein (Figure S7A). Using electron microscopy, we demonstrated a loss of homeostatic regulation in the ER in the *Jz-Perk*^*−/−*^ placentas, with ER cisternae dilated only in spongiotrophoblasts (Figure 4A, arrowheads). Protein glycosylation was also perturbed as shown by staining with *Datura stramonium* lectin (DSL). This binds to GlcNAc oligomers and anomalous glycoprotein aggregates/deposits accumulated exclusively in the Jz of the *Jz-Perk*^*−/−*^ placentas (Figure 4B, green arrows) and were clearly visible in electron micrographs of the Jz of the *Jz-Perk*^*−/−*^ placenta (Figure S7B, arrowheads). Furthermore, we detected aggregates showing positive staining with two other lectins (Concanavalin A, Con A, and *Pisum sativum* Agglutinin, PSA) which both recognize α-linked mannose, present either as part of a core oligosaccharide (Con A) or as an α-linked mannose-containing oligosaccharide (PSA, Figure S7C, arrows). This implies a potential change in glycan structures with enriched oligomannose of the glycoproteins, indicating mis-glycosylation, synthesized by the spongiotrophoblasts under ER stress. A similar pattern of lectin staining and dilated ER cisternae was also found in the Jz of *Eif2s1*^tm1Rjk^ mutant placentas, which also display ER stress exclusively in the Jz.^30^

In females housed under reduced oxygen, the litter size (ie the number of both live and dead fetuses) did not differ between females carrying either *Perk*^*fl/fl*^ or *Jz-Perk*^*−/−*^ litters (median(range): 7 (5-13) vs 7.5 (5-11)), Figure S7D). Females carrying *Jz-Perk*^−/−^ placentas had a non-significant change in resorption rate compared to females carrying control *Perk*^*fl/fl*^ placentas (13.3% increase, p = 0.131, Figure 4C). There was a trend to an increase in the weight of the *Jz-Perk*^*−/−*^ placentas (6.8% higher, p = 0.086) but no change in fetal weight (Figure 4D). The placental efficiency showed a non-significant reduction (8.0%, p = 0.228, Figure 4E). These results show that while placental ER stress *in vivo* causes intracellular aggregation of proteins in spongiotrophoblasts, it had little impact on placental growth, placental efficiency, and the frequency of embryonic loss.

### Perturbation of placental endocrine function causes maternal maladaptation in hepatic glucose metabolism and induces cellular stress in the liver

**Figure 5:**
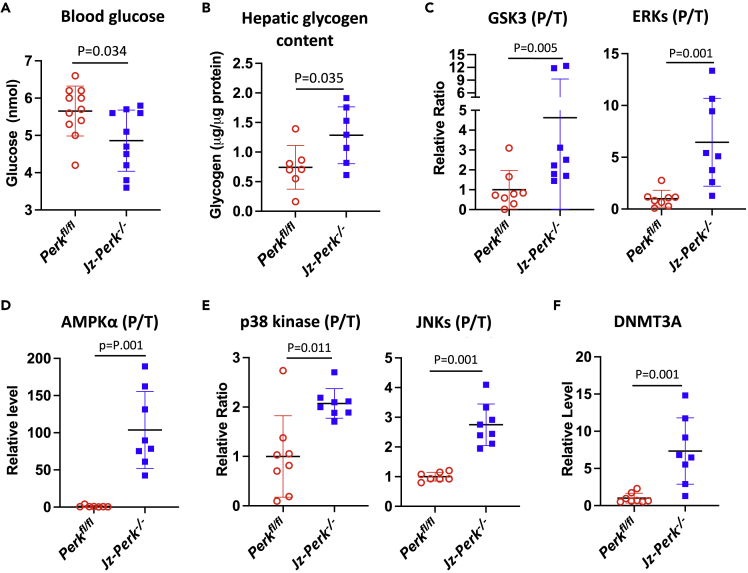
Jz-specific ER stress alters maternal blood glucose and induces hepatic metabolic dysfunction and cellular stress, and potentially facilitates epigenetic change (A) Maternal blood glucose concentration was measured by Glucometer at E18.5. Data are presented as mean ± SD, *Jz-Perk*^*−/−*^ = 10; *Perk*^*fl/fl*^ = 11. Unpaired t-test. (B) Glycogen content in maternal liver measured at E18.5 Data are presented as mean ± SD, *Jz-Perk*^*−/−*^; *Perk*^*fl/fl*^ both n = 7. Unpaired t-test. (C–E) Western blot analysis of maternal liver for kinases or proteins involved in hepatic insulin and metabolic signaling and cellular stress pathways. (C) GSK3s and ERKs; (D) AMPKα; (E) p38 kinase and JNKs. (F) Western blot analysis of DNA methyltransferase 3 alpha (DNMT3A) in maternal liver. (C–F) Data are presented as median with 95% Cl, n = 8. Mann-Whitney U test.

The liver plays a crucial role in maternal metabolism, including increasing glucose and lipid availability near term to support fetal growth and in preparation for lactation.^39^ We, therefore, tested whether the normal physiological adaptations occurred in the liver of pregnant females carrying litters of *Jz-Perk*^−/−^ placentas compared to *Perk*^*fl/fl*^ placentas. Maternal blood glucose concentration was measured and liver tissue harvested at E18.5. In pregnancies unaffected by ER stress (i.e. housed under atmospheric oxygen) there was no difference in maternal blood glucose concentration between the groups (Figure S6C). In contrast, under Jz ER stress-inducing conditions, maternal blood glucose concentration was reduced by 14.1% (p = 0.024) in females carrying *Jz-Perk*^−/−^ placentas compared to control *Perk*^*fl/fl*^ placentas (Figure 5A). Hepatic glycogen content increased by 73.3% (p = 0.035) (Figure 5B), suggesting potential maternal hepatic maladaptation in females carrying *Jz-Perk*^−/−^ placentas.

Both the AKT-GSK3 and ERK1/2 signaling pathways are central for the regulation of hepatic glycogen metabolism upon insulin or growth factor stimulation.^40^ There was a 4.6-fold (p = 0.005) and 6.5-fold (p = 0.001) increase in the ratio between phosphorylated and total glycogen synthase kinase 3 α/β (GSK3α/β) and extracellular-regulated kinases (ERK1/2) respectively in females carrying *Jz-Perk*^−/−^ placentas (Figures 5C, S8, and S9A). ERKs and GSK3s respectively are activated and inactivated by phosphorylation and this promotes hepatic glycogen synthesis.^40^ Phosphorylation of PDK1 and AKT, upstream kinases regulating GSK3 phosphorylation, was increased while there was no change in the phosphorylation status of 4E binding proteins (4EBP1) (Figure S8). This suggests that whatever signals the maternal liver is receiving, they are likely promoting glycogen synthesis rather than protein translation, cell growth, and proliferation. To conclude, these results indicate that loss of correct placental signals results in maternal liver maladaptation and disruption of glucose homeostasis during pregnancy.

Anomalous hepatic glucose metabolism could impact on the maternal hepatic energy supply. Therefore, the activity of the energy-sensing kinase AMP-activated protein kinase α (AMPKα) was investigated. Indeed, AMPKα phosphorylation was markedly elevated (over 100-fold, p = 0.001) in the liver of females carrying *Jz-Perk*^−/−^ placentas (Figures 5D and S9A). As expected, the energy deprivation was associated with cellular stress in the liver as indicated by a 2-fold (p = 0.011) and 2.7-fold (p = 0.001) increase of stress kinases, p38 kinase and c-Jun N-terminal kinases (JNKs) phosphorylation respectively (Figures 5E and S9A). Some markers of the unfolded protein response for mitochondria and ER were also increased significantly (Figure S9B). These differences between females carrying *Jz-Perk*^−/−^ and *Perk*^*fl/fl*^ placentas could not be accounted for by litter size (Figure S7D).

Finally, we also observed a 7.3-fold (p = 0.001) increase of DNA methyltransferase 3A (DNMT3A) in the liver of females carrying *Jz-Perk*^−/−^ placentas (Figures 5F and S10A). Other epigenetic regulators (DNMT1 and TET1) were undetectable in adult liver and MECP2 was unchanged in the two groups (Figure S10B). DNMT3A is crucial in facilitating *de novo* DNA methylation in somatic cells.^41^ This suggests the maladaptive changes induced in maternal organs during pregnancy can epigenetically alter the genome of affected cells. This could potentially increase the risk of developing metabolic diseases in later life and warrants further study.

### Placental proteins are aberrantly glycosylated in early-onset pre-eclampsia

**Figure 6:**
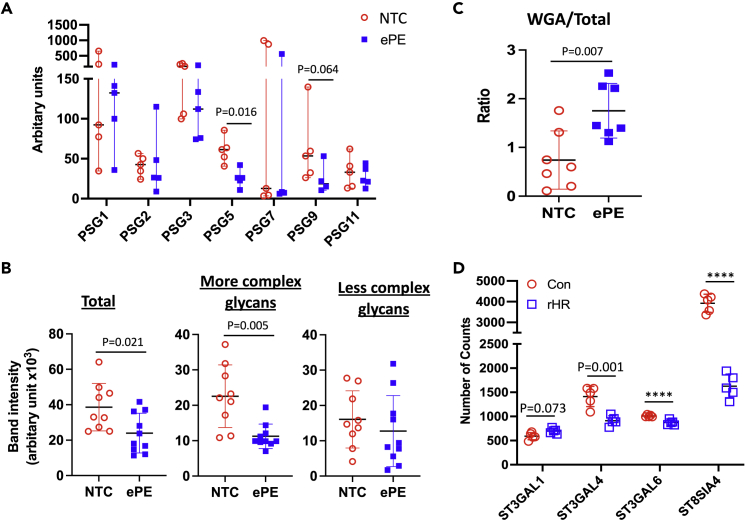
Mis-glycosylated PSG5 is present in the placenta and maternal circulation in patients who develop ePE (A) PSGs detected in maternal serum by quantitative TMT-LC-MS/MS analysis. Gestational age-matched albumin-depleted normotensive control (NC) and ePE serum samples were purified by WGA affinity chromatography before mass spectrometry. Levels of PSGs are plotted as a median with 95% Cl, n = 4-5. Mann Whitney U test. (B) PSG5 level and glycosylation profile in NC and ePE serum. Western blot band intensities corresponding to “More complex glycans” and “Less complex glycans” were quantified separately and are plotted together with “Total” which is the overall band intensity. Data are mean ± SD, n = 9-10. Unpaired t-test. (C) The ratio of WGA affinity-purified to total PSG5 in normotensive term control (NTC) and ePE placentas. The results in B-C are expressed as mean ± SD, n = 7-10. Unpaired t-test. (D) Sialyltransferases involved in N-glycan sialylation are down-regulated in repetitive hypoxia-reoxygenation (rHR)-treated BeWo cells. Cells were challenged with rHR (a 6 h cyclic pattern between 20% and 1% O_2_ concentration for 24 h). The number of normalized counts from RNA-Seq are plotted. Data are mean ± SD, n = 5. Paired t-test. ∗∗∗∗ indicates p < 0.0001.

Placental ER stress is a feature of early-onset pre-eclampsia^23^^,^^26^ so we next sought evidence that the ER stress-mediated protein mis-glycosylation occurs in this condition. We first investigated whether placentally derived proteins in the maternal circulation of patients with pre-eclampsia are mis-glycosylated. We collected gestational age-matched maternal serum from women who remained normotensive (normal controls, NC) or went on to subsequently develop early onset pre-eclampsia (ePE), with samples collected at 28 ± 1.9 wkGA (NC) and 28 ± 2.4 wkGA (ePE) mean ± SD, n = 5 in each group, Table S8). We hypothesized that changes in glycan structures illustrated in Figure 1A would alter their binding affinity to lectins. Therefore, to identify serum proteins with different glycosylation patterns we used wheat germ agglutinin (WGA) affinity chromatography to enrich glycoproteins before conducting isobaric Tandem Mass Tags (TMT)-labeled quantitative LC-MS/MS analysis. This lectin which binds the sugar N-acetyl-D-glucosamine (GlcNAc) with preferential binding to dimers and trimers (it also has some affinity to sialic acid). We identified 113 proteins present in 9 out of 10 samples with at least 2 different peptides detected (Table S9). Seven of these were members of the pregnancy-specific glycoprotein (PSG) family. The PSGs are highly abundant glycoproteins secreted by the placenta.^42^ It has been suggested that they may regulate maternal immune and vascular function and that they may have autocrine and paracrine functions.^43^ There was a considerable spread of the measured values of the circulating PSGs. Of note, PSG7 was either very high or very low (Figure 6A). This is consistent with the bimodal levels of *PSG7* mRNA in the term human placenta (Figure S11).^44^ However, the copy number of the genes in the PSG locus is highly variable and one PSG7 splice variant undergoes nonsense-mediated decay. These, and other mechanisms, likely contribute to the heterogeneity in the levels of PSG protein and mRNA.^43^ Circulating PSG5 and PSG9 were lower in the ePE sera (58%, p = 0.016 and 60%, p = 0.064 respectively) compared to NC sera, while other PSGs were unchanged (Figure 6A).

PSG5 has four predicted N-linked glycosylation sites at asparagine residues N104, N111, N175, and N210 (UniportKB-Q15238). After prolonged gel electrophoresis of serum proteins, we detected multiple immunoreactive PSG5 bands, the pattern differing between NC and ePE sera (Figure S12A). It should be noted that definitive determination of anti-PSG antibody specificity is challenging^43^ However, the antibody used here was selected as being targeted against a region of PSG5 that is not glycosylated (amino acids 287-336). The other most closely related PSGs (PSG3, PSG2, and PSG11) have 81.6%, 75.5%, and 63.3% identity respectively in this region. The level of these PSGs in WGA-purified serum was unchanged when using an antibody-independent assay (Figure 6A) suggesting that cross-reactivity, as might be seen in Figure S12C is unlikely. However, this possibility cannot be conclusively ruled out. The predicted molecular weight of non-glycosylated PSG5 is 38 kDa; however, the apparent molecular weights of the PSG5 bands ranged from ∼35 to ∼50 kDa, indicating the protein contains a range of glycans at its multiple glycosylation sites. Western blot analysis of serum PSG5 (without WGA-affinity purification) was consistent with the result obtained by mass spectrometry with serum PSG5 being lower in ePE (37.9%, p = 0.019). It is worth noting that the circulating placentally derived mis-glycosylated glycoproteins without “protective” sialic acid end cap and/or with high oligomannose are likely to be cleared from the circulation.^45^ However, analysis of the immunoreactive bands corresponding to proteins with more, or less, complex glycans showed that the biggest change was in the loss of PSG5 which has more complex glycan side chains, (50.2%, p = 0.002, Figure 6B). In contrast, the concentration of PSG5, which has less complex side chains, remained relatively constant.

To further investigate the mis-glycosylation of PSG5 as a result of placental ER stress, we collected placentas from women with ePE and normotensive term controls (Table S10). Western blot analysis showed multiple PSG5 bands indicating differences in glycosylation (Figure S12B). The bands with higher molecular weight were predominantly present in the NTCs (Figure S12B), consistent with the reduced complexity of PSG5 glycan we observed in ePE serum. The results using BeWo cells suggested that ER stress reduces sialylation and glycan complexity and that mis-glycosylation of PSG5 could expose more glycans with terminal N-acetylglucosamine (GlcNAc). Therefore, the same placental tissue lysates were subjected to WGA affinity chromatography to isolate PSG5-containing glycans with terminal GlcNAc prior Western blotting analysis (Figure S12C). To overcome the unavoidable difference in gestational age of placental samples between NTC (39 ± 0.8 weeks) and ePE (27 ± 2.1 wk) (Table S10) we calculated the ratio between WGA-enriched and total PSG5 in placental lysates. There was an ∼2-fold (p = 0.007) increase in the ratio (WGA/Total) of PSG5 in the ePE placentas (Figure 6C). These results confirm that ePE placentas that suffer from ER stress synthesize and secrete mis-glycosylated glycoproteins into the maternal circulation.

Due to the importance of sialylation for the stability of circulating glycoproteins, we investigated gene expression of all fifteen human sialyltransferases involved in the sialylation of both N- and O-linked glycosylation^31^ by quantitative RT-PCR (RT-qPCR) in NTC and ePE placentas (9 per group). Five of the mRNAs were significantly reduced (*ST3GAL1* ↓ 22%, p = 0.027, *ST3GAL4* ↓ 52%, p = 0.042, *ST3GAL6* ↓ 30%, p = 0.003, *ST8SIA3* ↓ 57% (p = 0.027), *ST8SIA4* ↓ 44%, p = 0.032) while two of the mRNAs show a trend (*ST3GAL3* ↓ 27%, p = 0.053) and no change (*ST3GAL5*, p = 0.297) respectively (Figure S13A). The remaining eight (*ST3GAL2*, *ST6GAL1*, *ST6GALNAC1*, *ST6GALNAC2*, *ST6GALNAC4*, *ST8SIA1* and *ST8SIA2 and ST8SIA5*) had very low expression (>34 PCR cycles) in the placenta. Western blotting showed ST3GAL6 was reduced by 56% (p = 0.016) (Figure S13B). However, all these data are unavoidably confounded by gestational age differences at the time of placental collection. Therefore, we investigated sialyltransferase expression in RNA-Seq datasets from BeWo cells after 24 h repetitive hypoxia-reoxygenation (HR) challenge (HR-induced injury is a potent inducer of ER stress and is central to placental pathophysiology in ePE^26^^,^^46^). We have demonstrated that this rHR model recapitulates the changes in ER and mitochondrial UPR signaling pathways observed in ePE placentas.^26^^,^^47^ The expression of three of the five sialyltransferases down-regulated in the ePE placentas was significantly reduced in rHR-treated cells - *ST3GAL4* (↓35.5%, p = 0.001), *ST3GAL6* (↓13.5%, p = 0.005), and *ST8SIA4* (↓58.5%, p < 0.0001) while for *ST3GAL1* there was a non-significant reduction (↓7.4%, p = 0.073) (Figure 6D). *ST8SIA3* was only expressed a very low level with fewer than 2 counts. This suggests that placental stress in ePE is the likely cause of down-regulation of these three sialyltransferases.

Reduced expression of sialyltransferases prevents or reduces the correct addition of the sialic acid “protective shield” on these proteins. This loss would compromise their circulating half-life and possibly their ability to correctly signal to the maternal tissues/organs. The reduction of maternal serum PSG5 concentrations while placental WGA-binding PSG5 is elevated in ePE is consistent with this rationale. Indeed, glycoproteins with glycans carrying terminal GlcNAc are selectively cleared from the circulation in both humans and monkeys.^48^ In summary, our results reveal that the glycosylation profile of placentally derived glycoproteins is altered in placentas from cases of early-onset pre-eclampsia.

## Discussion

**Figure 7:**
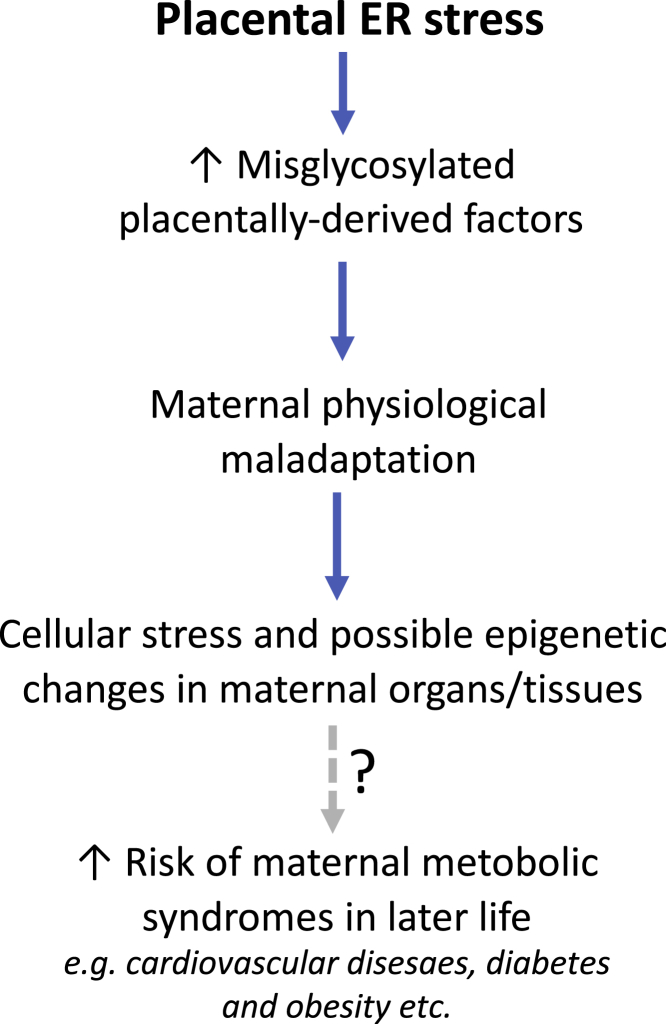
Summary diagram illustrating potential pathways and consequences of placental ER stress induced mis-glycosylation of placentally derived factors and risk of maternal metabolic disorders in later life

During pregnancy, placental hormones modulate maternal metabolism to support fetal growth and lactation. In this study, we hypothesized that placental ER stress causes aberrant glycosylation and loss of functional activity of some of the signaling hormones and this compromises maternal adaptations to pregnancy. Our new transgenic animal model of ER stress specific to the placental endocrine zone demonstrated an association between the alteration of normal placental signals and compromised maternal hepatic glucose metabolism. The maladaptation led to increased cellular stress in the maternal liver and increased hepatic DNA methyltransferase (DNMT3A) expression, which in turn could result in epigenetic changes (Figure 7). Furthermore, we show mis-glycosylation and reduced concentrations of secreted placental glycoproteins in pregnancies complicated by early-onset pre-eclampsia. Our results, therefore, provide the first potential mechanistic link between pregnancy complications associated with placental ER stress and adverse maternal long-term health.

N-Glycan biosynthesis is initiated in the ER and completed and matured in the Golgi apparatus through the actions of a series of glycotransferases and glycosidases.^49^ For the biosynthesis, a mature N-glycan precursor Glc_3_Man_9_GlcNAc_2_-P-P-Dol is first synthesized in the ER before transfer to the asparagine residue in receptive Asn-X-Ser/Thr sequons in newly synthesized proteins. A trimming process on this 14-sugar glycan follows the formation of Man_8_GlcNAc_2_-Asn before the translocation of the protein to the Golgi apparatus for further glycan modification. N-glycan biosynthesis is only completed and matured by “capping” reactions, in which sialic acids, fucose (Fuc), galactose (Gal), and/or N-acetylglycosamine (GlcNAc) are added to the complex N-glycan branches. Capping sugars facilitate the presentation of terminal structures to lectins and antibodies.^49^ Our RNA-Seq result revealed that ER stress alters the expression of 66 genes (FC > 1.5) involved in protein glycosylation (GO:00006486) and affecting almost every step of N-glycan biosynthesis (Figure S4). GSEA further revealed the potential alteration of the trimming process of the precursor 14-sugar glycan by ER stress. As the precursor glycan is central for subsequent glycan oligomannose and complex glycan formation, this finding suggests that ER stress not only induces mis-glycosylation, but may also reduce overall protein glycosylation. This is supported by increased RNA from genes involved in protein deglycosylation (Figure S3B). Indeed, we observed a reduced degree of glycosylation of hCGβ and VEGFA under Tg treatment. ER stress-mediated alterations of glycan structures have been reported using genetic manipulation or the ER stress inducer thapsigargin (Tg),^22^ which also revealed that the induced glycosylation change affects both secreted and membrane protein glycosylation.^22^ This finding is consistent with changes in lectin staining in placentas from cases of pre-eclampsia,^50^^,^^51^ where ER stress-mediated alteration of placenta protein glycosylation may affect the activity of placentally derived proteins and the glycans arrayed on membrane receptors, carriers, and transporters on the syncytiotrophoblast and endothelial cells. These could have profound effects on “self-nonself” discrimination during pregnancy, receipt of correct maternal signals, and nutrient exchange. Interruption of this two-way communication between the mother and placenta could also have significant effects on maternal physiological adaptations and placental function, resulting in poor pregnancy outcomes.

The composition of the glycocalyx covering the placenta is changed in cases of pre-eclampsia,^50^^,^^51^ with an increase in oligomannose and a decrease in sialic acids in both syncytiotrophoblast and fetal endothelial cells.^51^ Reduced structures with α-2,3-linked sialic acid in placentas from ePE is consistent with the downregulation of sialyltransferases ST3GAL4 and ST3GAL6 and exposes glycans with terminal galactose. On the other hand, the increase of WGA-isolated placental PSG5 also indicates that the ePE placenta synthesize glycoproteins containing high level of glycans with terminal GlcNAc, rather than sialic acid. Sialylation protects glycoproteins from glycosidases and proteases,^18^ thus increasing their circulating half-life. In the liver, asialoglycoprotein receptors (Asgrs) on hepatocytes and the mannose receptor (ManR) on sinusoidal endothelial cells and Kupffer cells are two conserved receptors involved in the clearance of circulating glycoproteins with exposed terminal galactose structures due to lack of sialic acid end-caps and containing high mannose N-glycan respectively.^52^ These receptors also play a central role in the clearance of placentally derived factors with aberrant glycosylation.^45^ Additionally, glycoproteins with terminal GlcNAc can also be selectively cleared from the circulation by the clearance receptor(s), which has not yet to be identified.^48^ However, an *in vitro* study shows the glycoproteins with terminal GlcNAc can bind to ManR^53^ and this increased clearance could explain why the concentration of PSG5 which contains high terminal GlcNAc, was reduced in ePE serum. Crucially, our results demonstrated that the glycosylation change in the ePE placenta is consistent with ER stress. Using the *Jz-Perk*^*−/−*^ model, we further showed that correct placental signals are necessary for normal maternal hepatic glucose metabolism and avoidance of cellular stresses induced by the physiological changes during pregnancy.

Mounting epidemiological evidence links pregnancy complications to an increased maternal risk of metabolic disease in later life.^5^^,^^6^^,^^7^ Lack of the correct placental signals that normally induce changes in maternal organs to meet fetal demands could overwhelm homeostatic mechanisms and induce cellular stress, thereby causing irreversible damage. The increase in hepatic *Dnmt3a* mRNA, encoding a DNA methyltransferase, may implicate *de novo* DNA methylation in the liver cells under stress.^41^ A change in liver DNA methylation of genes relevant to the development of diabetes is seen in patients with type II diabetes.^54^ A *post-partum* mouse study will be necessary to test links between the genome-wide DNA methylation pattern in livers from females carrying *Jz-Perk*^*−/−*^ placentas and signs of diabetes in later life.

The placenta also releases other factors (cytokines, chemokines, and so forth) not analyzed in the course of this work. It is likely that the release of these may also be altered by placental ER stress. These could also affect maternal adaptation so would be an area for future study.

To conclude, we show how placental stress can lead to maternal physiological maladaptation to pregnancy by causing aberrant glycosylation of placentally derived factors. Alleviating this stress, therefore, has the potential to improve long-term maternal health. We recently showed that tauroursodeoxycholic acid (TUDCA), a biliary salt, greatly reduces chronic placental ER stress.^55^ As the related ursodeoxycholic acid (UDCA) has already been used in the treatment of intrahepatic cholestasis during pregnancy,^56^ interventions based on this chaperone are worth further study.

### Limitations of the study

This study has four main limitations; i) We generated several lines of evidence, albeit indirect, that show placental cells when exposed to ER stress secrete aberrantly glycosylated proteins and that misglycosylated protein looses its normal biological activity. Nevertheless, our results are consistent with the study by Wong et al. ^22^ but direct evidence would be stronger. ii) In the placental-specific ER stress (*Jz-Perk*^*−/−*^) animal model, we had to use mild hypoxia to exacerbate ER stress in the placenta. Although *Perk*^*fl/fl*^ animals were also exposed to hypoxia, we cannot exclude possible interference of hypoxia on maternal physiology. To overcome this we are developing another animal model with placental ER stress without the requirement for an additional stressor. iii) The placental-specific ER stress (*Jz-Perk*^*−/−*^) animal model has defective maternal mal-adaptation of hepatic glucose metabolism. However, the detail of which placental protein(s) mediate this and mechanism underlying this are not known. iv) While we demonstrated the misglycosylation of placental proteins in human pregnancy, it is not known whether the metabolic maladaptation we observed in mice also occurs in humans.

## STAR★Methods

### Key resources table

**Table:** 

REAGENT or RESOURCE	SOURCE	IDENTIFIER
**Antibodies**		

β-actin	Sigma-Aldrich	Cat#A2228; RRID:AB_476697
Phospho-VEGF Receptor 2 (Tyr951)	Cell Signaling Technology	Cat#2476; RRID:AB_331367
VEGFR2	Cell Signaling Technology	Cat#2479; RRID:AB_2212507
Phospho-GSK-3α/β (Ser21/9)	Cell Signaling Technology	Cat#9331; RRID:AB_329830
GSK-3β	Cell Signaling Technology	Cat#9315; RRID:AB_490890
Phospho-p44/42 MAPK (Erk1/2) (Thr202/Tyr204)	Cell Signaling Technology	Cat#4370; RRID:AB_2315112
p44/42 MAPK (Erk1/2)	Cell Signaling Technology	Cat#9102; RRID:AB_330745
Phospho-p38 MAPK (Thr180/Tyr182)	Cell Signaling Technology	Cat#9211; RRID:AB_331641
p38 MAPK	Cell Signaling Technology	Cat#9212; RRID:AB_330713
Phospho-SAPK/JNK (Thr183/Tyr185)	Cell Signaling Technology	Cat#9251; RRID:AB_331659
SAPK/JNK	Cell Signaling Technology	Cat#9252; RRID:AB_2250373
Phospho-AMPKα (Thr172)	Cell Signaling Technology	Cat#2531; RRID:AB_330330
AMPKα	Cell Signaling Technology	Cat#2532; RRID:AB_330331
PSG5	Abcam	Cat#ab97940; RRID:AB_10696266
VEGFA	Abcam	Cat#ab46154; RRID:AB_2212642
PLGF	Abcam	Cat#ab9542; RRID:AB_307330
hCG beta	Abcam	Cat#ab70884; RRID:AB_1280813

**Biological samples**		

Human Placental Tissues	Uppsala University; Prof Olovsson’s biobank (Wikstrom et al. 2009^57^)	
Human Serum Samples	King College London; Prof Chappell’s biobank (Bramham et al. 2016^58^)	

**Chemicals, peptides, and recombinant proteins**		

Proteinase K	Sigma-Aldrich	Cat#P6556
Thapsigargin	Sigma-Aldrich	Cat#T9033
Recombinant Vegfa	Sigma-Aldrich	Cat#V4512
Concanavalin A-biotinylated lectin	Vector Laboratories	Cat#B-1005
Pisum Sativum Agglutinin-biotinylated lectin	Vector Laboratories	Cat#B-1055
*Datura stramonium* Agglutinin-biotinylated lectin	Vector Laboratories	Cat#B-1185
Avidin peroxidase	Sigma-Aldrich	Cat#A3151
3′3′diaminobenzidine tetrahydrochloride dihydrate	Sigma-Aldrich	Cat#26189-0
Trypsin from porcine pancreas	Sigma-Aldrich	Cat#T7409
Propylene oxide	TAAB Laboratories Equipment	Cat#P021
TAAB epoxy resin	TAAB Laboratories Equipment	Cat#T004
EM grade glutaraldehyde	Agar Scientific Ltd	Cat#R1010
Sodium cacodylate	TAAB Laboratories Equipment	Cat#S006
PIPES, disodium salt	Sigma-Aldrich	Cat#P3768

**Critical commercial assays**		

Colorimetric glycogen Assay kit	Abcam	Cat#ab169558
Direct PCR Lysis Reagent	Bioquote Ltd	Cat#102-T (tissues) Cat#402-E (ear biopsy)
Pierce Glycoprotein Isolation Kit, WGA	ThermoFisher Scientific	Cat#89805
ProteoPrep® Blue Albumin & IgG Depletion Kit	Sigma-Aldrich	Cat#PROTBA-1KT
Bicinchoninic Acid Kit for Protein Determination	Sigma-Aldrich	Cat#BCA1
Quick Start™ Bradford Protein Assay Kit	Bio-Rad	Cat# 5000201
TMT 10plex Mass Tag Labeling Kits and Reagents	ThermoFisher Scientific	Cat#90110
RNeasy Mini Kit	Qiagen	Cat#74104
RNeasy Plus Universal Mini Kit	Qiagen	Cat#73404
SuperScript III Reverse Transcriptase	ThermoFisher Scientific	Cat#18080044
Taq DNA polymerase	ThermoFisher Scientific	Cat#10342020
SYBR® Green JumpStart™ Taq ReadyMix™	Sigma-Aldrich	Cat#S4438
Absolute qPCR ROX Mix	ThermoFisher Scientific	Cat#AB-1138/B
Remove-iT® PNGase F	New England Biolabs	Cat#P0706
Chromis DGE Minimal Labeling Kit	Cyanagen, Bologna, Italy	

**Deposited data**		

Raw RNAseq data	This paper	E-MTAB-10943, https://www.ebi.ac.uk/arrayexpress/experiments/E-MTAB-10943/
Analyzed data	This paper	Tables S1, S2, S3, S4, S5, S6 and S7

**Experimental models: Cell lines**		

BeWo cells	ATCC	CCL-98
Primary *Eif2s1*^tm1RjK^ mouse embryonic fibroblasts	This paper	
Human umbilical vascular endothelial cells	Cindrova-Davies et al. 2011 (https://doi.org/10.1093/cvr/cvq346)	

**Experimental models: Organisms/strains**		

Wild type mice, C57Bl/6	Charles River Laboratories	MGI Cat# 2159769; RRID:MGI:2159769
*Eif2s1*^tm1RjK^ mutant mice	Gift from Prof Randal Kaufman	MGI:2388264; RRID:IMSR_JAX:017601
*Eif2ak3*^*tm1*.*2Drc*^ (*Perk*^*fl/fl*^)	Gift from Prof Douglas Cavener	MGI:5502572; RRID:IMSR_JAX:023066
*Tg(Tpbpa-cre*,*EGFP)5Jcc*	Gift from Dr Miguel Constancia	MGI:5287872

**Oligonucleotides**		

Primers: *Eif2s1*^tm1RjK^; Forward: 5′-ATTCTTCTTAGTGAATTGGCCAGACGACGTATC-3′; Reverse: 5′-GATACGTCGTCTGGCCAATTCACTAAGAAGAATG-3′	This paper	N/A
Primers: *Eif2ak3*^*tm1*.*2Drc*^; Forward: 5′- CACTCTGGCTTTCACTCCTCACAG-3′; Reverse: 5′-GTCTTACAAAAAGGAGGAAGGTGGA-3′	This paper	N/A
Primers: *Tpbpa-Cre*; Forward: 5′TCCAGTGACAGTCTTGATCCTTAAT-3′; Reverse: 5′-AAATTTTGGTGTACGGTCAGTAAAT-3′	This paper	N/A
Primers: *ST3GAL1*; Forward: 5′-TTCTGAAAAGGAGGGAGTTC-3′; Reverse: 5′-GGAGTAGTTCAGGAAGAAGG-3′	This paper	N/A
Primers: *ST3GAL3*; Forward: 5′-CAGTGGTTCTTTCCTTTGAC-3′; Reverse: 5′-TAATTCAGCAGGCAGTTTAG-3′	This paper	N/A
Primers: *ST3GAL4*; Forward: 5′-GGGATGTCAATCCTAAACAG-3′; Reverse: 5′-CTTCTGCTTAATCTTCCGTG-3′	This paper	N/A
Primers: *ST3GAL6*; Forward: 5′-GAGGTTTCATCAGTTTCACC-3′; Reverse: 5′-AGGGCAAATCAAACTTATCG-3′	This paper	N/A
Primers: *ST8SIA3*; Forward: 5′-CATTACGAATTCTCTCACCC-3′; Reverse: 5′-CCTTTGATGTAAAAACGCTG-3′	This paper	N/A
Primers: *ST8SIA4*; Forward: 5′-AAGGTGTAATCTAGCTCCTG-3′; Reverse: 5′-GCTCTTTGTACAACTGATGG-3′	This paper	N/A
Primers: *TBP*; Forward: 5′-GTGGGGAGCTGTGATGTGA-3′; Reverse: 5′AATAAGGAGAACAATTCTGGTTTG-3′	This paper	N/A
Primers: *18S*; Forward: 5′-GTAACCCGTTGAACCCCATT-3′; Reverse: 5′-CCATCCAATCGGTAGTAGCG-3′	This paper	N/A
Primers: *Gcm1*; Forward: 5′-CATCTACAGCTCGGACGACA-3′; Reverse: 5′-CCTTCCTCTGTGGAGCAGTC-3′	This paper	N/A
Primers: *Vegfa*; Forward: 5′-TGCGGATCAAACCTCACCAA-3’; Reverse: 5′-CGCCTTGGCTTGTCACAT-3′	This paper	N/A
**TaqMan Assays**		
*Tpbpa*	ThermoFisher Scientific	(Mm00493788_g1)
*Hprt*	ThermoFisher Scientific	(Mm01545399_m1)

**Software and algorithms**		

mageMaster2D, DeCyder 5.02 version software	GE Healthcare	
Proteome Discoverer v2.1	ThermoFisher Scientific	
Mascot v2.6	Matrix Science	
R package MSnbase	Gatto and Lilley 2012^59^	
Limma Package		https://bioconductor.org/packages/release/bioc/html/limma.html
Data Explorer 4.9 Software	Applied Biosystem	
GlycoWorkBench	Ceroni et al. 2008^60^	
Nextflow, nf-core/rnaseq	Ewels et al. 2019^61^	https://nf-co.re/rnaseq
R package DESeq2 v1.28.0	Love et al. 2014^62^	https://bioconductor.org/packages/release/bioc/html/DESeq2.html
R package gprofiler2 v0.2.1	Kolberg et al. 2020^63^	https://cran.r-project.org/web/packages/gprofiler2/index.html
GESA	Subramanian et al. 2005^64^	https://www.gsea-msigdb.org/gsea/index.jsp

**Other**		

Nextflow corresponding software and versions	This paper	Table S15
Repository of custom code	This paper	Zenodo, https://doi.org/10.5281/zenodo.7373310

### Resource availability

#### Lead contact

Further information and requests for resources and reagents should be directed to and will be fulfilled by the lead contact, D. Stephen Charnock-Jones (dscj1@cam.ac.uk).

#### Materials availability

This study did not generate new unique reagents.

### Experimental model and subject details

#### Placental tissue collection

Patients were recruited at the University Hospital, Uppsala, Sweden. All placental samples were obtained with local ethical permission and the patients’ informed written consent. The detailed criteria for recruitment of patients have been described previously.^57^ Briefly, pre-eclampsia was defined as new-onset hypertension (≥140/90 mmHg) observed on at least two separate occasions, 6 h or more apart, combined with proteinuria (a 24 h urine sample showing ≥300 mg/24 h). The control group was from healthy normotensive term patients that displayed no abnormalities on routine scans. Women with essential hypertension, diabetes mellitus or pre-existing renal disease were excluded. Both early-onset PE (<34 weeks) and normotensive term control placentae were obtained from elective, non-laboured caesarean deliveries. For each placenta, four to six small pieces of tissue from separate lobules were rinsed in saline, blotted dry and snap-frozen in liquid nitrogen within 10 min of delivery; the samples were stored at −80°C. Patient clinical characteristics are presented in Table S10.

#### Maternal serum collection

The criteria for patient selection have been described previously.^58^ Briefly, women were prospectively enrolled at 2 London academic health science centers (Imperial College and King’s Health Partners) between June 2009 and September 2013. Ethical approval was provided by the National Research Ethics Service (11/LO/1776), and the study was performed in accordance with the guidelines of the Declaration of Helsinki. Venous blood samples were taken up to 4 times during pregnancy, and serum and plasma were stored at −80°C. Maternal and perinatal outcome data were obtained by case note review after delivery. Definitions for study entry and outcomes are based on International Society of Study of Hypertension in Pregnancy guidelines.^65^ Patient clinical characteristics are listed in Table S8.

#### Mice

All animal work was performed under the authority granted by the Animals (Scientific Procedures) Act 1986 Amendment Regulations 2012 and following ethical review by the University of Cambridge Animal Welfare and Ethical Review Body. Mice were housed in M3 conventional cages (NKP, UK), at 55% humidity and at 21°C with a 12 h light cycle. C57Bl/6 mice were purchased from Charles River Laboratories and bred in-house. Derivation and characterisation of the *Eif2s1*^tm1RjK^ (MGI:2388264), *Eif2ak3*^*tm1*.*2Drc*^ (*Perk*^*fl/fl*^) (MGI:5502572) and *Tg(Tpbpa-cre*,*EGFP)5Jcc* (MGI:5287872) lines have been described.^29^^,^^66^^,^^67^ These mice were gifted by Prof Randal Kaufman (Sanford-Burnham-Prebys Medical Discovery Institute, US), Prof Douglas Cavener (Pennsylvania State University, US) and Dr Miguel Constancia (University of Cambridge, UK) respectively. Pregnant females were identified by the presence of a vaginal plug, with noon of the day the plug being defined as E0.5. A junctional zone-specific *Perk* knockout, (*Jz-Perk*^*−/−*^) was generated using *Floxed Perk* (*Perk*^*fl/fl*^) and *Tpbpa-Cre*^*+/+*^ mice. The *Perk*^*fl/fl*^ females were crossed either with *Perk*^*fl/fl*^ males or with *Perk*^*fl/fl*^.*Tpbpa-Cre*^*+/+*^ males to generate *Perk*^*fl/fl*^ (control) and *Jz-Perk*^*−/−*^ (experimental) litters respectively. Animals were housed in 13% oxygen from E0.5 inside a hypoxia chamber, which combined a PVC isolator (PFI Plastics Ltd, Keynes, UK) with a nitrogen generator (N2MID60; Dominick Hunter Ltd, Warwick, UK).

### Method details

#### Genotyping of transgenic animals

For genotyping, an ear biopsy or a small piece of embryonic tissue was used. The tissue was lysed in Direct PCR Lysis Reagent (102-T for tissues or 402-E for ear biopsies, Bioquote Ltd, UK) containing 1 mg/mL proteinase K (P6556, Sigma UK) at 55°C for 2 h followed by 85°C for 45 min. 1 μL was used for PCR and PCR products were resolved at 2% agarose gel. For primer sequences, see Table S11.

#### Cell and tissue culture

All culture reagents were purchased from ThermoFisher Scientific, UK unless otherwise stated.

#### Jz explant culture

The placenta from E12.5 was dissected into decidua, junctional and labyrinth layers. The junctional layer was cut into 1 mm^3^ pieces and placed on a 40 μm cell strainer submerged in a 6 well plate. Jz explants were cultured for 48 h in a humidified 37°C incubator at 10% O_2_ in defined serum-free medium comprising: advanced DMEM/F12 reduced serum medium (12634), 1X GlutaMax (35050061), 1X penicillin/streptomycin (15140-122), 0.5X B27 supplement (17504044). Thapsigargin (100 nM, Sigma, UK) was added to some cultures. Conditioned medium was harvested and centrifuged at 4000 rpm (1771 g) for 30 min at 4°C to remove tissue debris before concentration using Vivaspin (5000 MWCO) centrifugal concentrator (Vivaproducts Inc, USA) according to manufacturer’s instructions. Protein concentration was determined using the BCA (Sigma, UK).

#### Mouse embryonic fibroblast isolation

Mouse embryos were collected at E12.5. The remainder of the embryo was incubated with 0.05% Trypsin/EDTA (11580626) overnight at 4°C. The Trypsin was replaced with 1 mL of fresh 0.05% Trypsin/EDTA and incubated for 15 min at 37°C with occasionally swirling. An equal amount of DMEM (41966) containing 10% FBS (10270106), 1X penicillin/streptomycin was added and tissue dissociated by gentle pipetting. The suspension was sieved through a 40 μm mesh to remove all aggregates before centrifugation. The pellet was resuspended in 10% FBS containing DMEM medium and the cells were cultured in a humidified 37°C incubator with 5% CO_2_.

#### BeWo cells

Human choriocarcinoma BeWo cells (ATCC, CCL-98) were cultured in modified DMEM/F12 medium containing 5.5 mM glucose, 10% FBS and 1X penicillin/streptomycin in a humidified 37°C incubator with 5% CO_2_. For all experiments and glycomic analysis cells were rinsed with serum free medium and then cultured for 24 h with Tg (100 nM) or vehicle (0.1% DMSO) in serum free medium. Conditioned medium was harvested, debris removed by centrifugation and stored at −80°C until use. Cells were not syncytialised as this can be somewhat variable and this would introduce experimental noise.

#### VEGFA stimulated VEGFR2 phosphorylation

Human umbilical vein endothelial cells (HUVECs) were kindly provided by Dr Cindrova-Davies (University of Cambridge).^68^ To assay VEGFA activity, HUVECs were pre-treated with serum-free medium for 6 h. The cells were then incubated with conditioned media, medium+25 ng/mL recombinant VEGFA (Sigma-Aldrich, UK), or medium alone for 10 min. HUVEC were harvested as described below and lysates stored at −80°C until Western blot analysis of the phosphorylation status of VEGFR2.

#### Western blotting

Western blotting was performed as previously described.^47^ Details of all antibodies are provided in Table S12.

#### Glycoprotein enrichment by lectin affinity chromatography, WGA

Glycoproteins were enriched from placental lysates or albumin-depleted sera using the Pierce Glycoprotein Isolation Kit, WGA (89805, ThermoFisher Scientific, UK) according to manufacturer’s instruction. The glycoproteins were eluted directly either with 1X gel loading buffer and boiled at 70°C for 10 min before Western blot analysis or with elution buffer for proteomic analysis.

#### Proteomic analysis

##### Two-dimension fluorescence Difference Gel Electrophoresis (2-D DIGE) of Jz explant conditioned media

DIGE and MS analyses of conditioned media harvested from the Jz explants were carried out in the Cambridge Center for Proteomics.^69^ Equal amounts of protein were mixed with Chaps/Thiourea buffer containing 6 M Urea, 2 M Thiourea, 4% Chaps, 10 mM Tris (pH 8), vortexed and sonicated to solubilise. pH was adjusted to pH 8.5 with 50 mM NaOH. Samples were quantified using Quick Start™ Bradford (Bio-Rad). Samples were then labeled with Cy5 dye and pooled with Cy3 dye according to the manufacturer’s instructions using Chromis DGE Minimal Labeling Kit (Cyanagen, Bologna, Italy) and incubated for 30 min. The reaction was quenched by adding 10 mM lysine and incubated for 10 min.

For IEF, the sample was soaked on 3–10NL Immobiline Dry Strip Gel (GE Healthcare). First dimension IEF focusing was performed with the Protean i12 IEF Cell (Bio-Rad) using the following parameters: 20V for 10 h, 500V for 1 h, 1000V for 1 h, 8000V until 40000 Vhrs was achieved. After IEF, the strips were equilibrated in a buffer containing 100 mM Tris (pH 6.8), 30% glycerol, 6 M Urea, 2% SDS and 1% DTT (freshly added) for 15 min following by 15 min in a buffer using the same composition except that 1% DDT was replaced with iodoacetamide 2.5%. The second dimension was then resolved in 12% SDS-polyacrylamide gel.

Gels were scanned using a Typhoon 9400 laser scanner (GE Healthcare) and analyzed using mageMaster2D, DeCyder 5.02 version software (GE Healthcare). Differential spots were identified, one of the gels silver stained and the spots of interest matched from the DeCyder image of the same gel to the silver stained image. Spots were cut manually and destained, reduced, alkylated and digested prior to LC-MS/MS analysis (see below).

##### TMT isobaric mass tags labelling of serum proteins

Serum albumin was depleted from both NC and ePE sera using ProteoPrep® Blue Albumin & IgG Depletion Kit (PROTBA-1KT, Sigma) following manufacturer’s instructions except tris-buffered saline (TBS) was used as equilibrium buffer. Protein concentration in albumin-depleted serum sample was measured using the BCA.

Glycoproteins were isolated from 1 mg albumin-depleted serum using Pierce™ Glycoprotein Isolation Kit, WGA. N-glycans were subsequently removed from the glycoproteins by enzymatic digestion with Peptide N-glycosidase. Briefly, the glycoproteins were precipitated using Isopropanol:Chloroform (4:1 ratio). After centrifugation at 12,000 g, the pellet was rinsed with 70% ethanol, air-dried and resuspended in Glycobuffer 2. Remove-iT® PNGase F (P0706, New England Biolabs) was used to remove glycans. After digestion, PNGase F was removed with Chitin Magnetic Beads (E8036, New England Biolabs) following manufacturer’s instructions.

The deglycosylated serum proteins were TMT labeled according to the manufacturer’s protocol (https://www.thermofisher.com/order/catalog/product/90110). 20 μg of each digested protein sample was labeled with 10 TMT tags using TMT 10plex Mass Tag Labeling Kits and Reagents (90110, Thermo Scientific). Post-labelling, samples were combined and cleaned on Sep-Pak C18 cartridge (87784, Thermo Scientific), dried and dissolved in 0.1% formic acid and placed in the LC autosampler.

##### Liquid chromatography and mass spectrometry (LC-MS/MS)

LC-MS/MS experiments were performed using a Dionex Ultimate 3000 RSLC nanoUPLC system and a Lumos Orbitrap mass spectrometer. Peptides were loaded onto a pre-column from the Ultimate 3000 auto-sampler with 0.1% formic acid for 3 min. Separation of peptides was performed by C18 reverse-phase chromatography using a reverse-phase nano Easy-spray column. The linear gradient employed was 2–40% B in 93 min. (Total LC run time was 120 min including a high organic wash step and column re-equilibration).

Eluted peptides from the C18 column LC eluant were sprayed into the mass spectrometer by means of an Easy-Spray source. All m/z values of eluting peptide ions were measured in an Orbitrap mass analyser. The top 10 most abundant fragment ions from each MS/MS event were then selected for a further stage of fragmentation by Synchronous Precursor Selection (SPS) MS3^70^ in the HCD high energy collision cell using HCD (High energy Collisional Dissociation, (NCE: 65%). The m/z values and relative abundances of each reporter ion and all fragments (mass range from 100-500 Da) in each MS3 step were measured in the Orbitrap analyser. All equipment and apparatus are from ThermoFisher Scientific.

##### Data analysis

Proteome Discoverer v2.1 (ThermoFisher Scientific) and Mascot v2.6 (Matrix Science) were used to process raw data files. Data were aligned with the UniProt human database, the common repository of adventitious proteins (cRAP) v1.0. Protein identification allowed an MS tolerance of ± 10 ppm and an MS/MS tolerance of ± 0.8 Da ppm along with permission of up to 2 missed tryptic cleavages. Quantification was achieved by calculating the sum of centroided reporter ions within a ± 2 millimass unit (mmu) window around the expected m/z for each of the four TMT reporter ions.

All comparative analyses were performed with the R statistical language. The R package MSnbase^59^ was used for processing proteomics data. Briefly, this entailed missing value removal (instances where a protein was identified but not quantified in all channels were rejected from further analysis), log2-transformation of the raw data, followed by sample normalization, utilizing the ‘diff.median’ method in Msnbase (this translates all samples columns so that they all match the grand median). Protein differential abundance was evaluated using the Limma package. Differences in protein abundance were statistically determined using the Student’s t-test with variances moderated by Limma’s empirical Bayes method. p values were adjusted for multiple testing by the Benjamini Hochberg method.

#### Glycomic analysis by MALDI-TOF MS

Glycan analysis of concentrated serum-free BeWo cell conditioned medium (reduced from 20 to 0.5 mL) was performed using established methodology as previously described (Vivaspin 20, MWCO 5 kDa centrifugal concentrator, Sartorius, UK).^71^ In brief, N-glycans were released from glycoproteins in the conditioned medium by digestion with PNGase-F enzyme at 37°C for 24 h. The free N-glycans were purified by C18-Sep-Pak (Waters, WAT051910) chromatography and permethylated. Permethylated samples were then purified by C18-Sep-Pak by stepwise elution with 15%, 35%, 50% and 75% acetonitrile in water.

Permethylated samples were dissolved in methanol, and 1 μL of sample was premixed with 1 μL of matrix (10 mg/mL 3,4-diami-nobenzophenone in 75% (v/v) aqueous acetonitrile) and spotted onto a MALDI-target plate and air-dried. The N-glycans were then analysed by matrix-assisted laser desorption ionisation-time of flight (MALDI-TOF MS and MALDI-TOF/TOF MS/ MS) mass spectrometry and the data were acquired using a 4800 MALDI-TOF/TOF (Applied Biosystems) mass spectrometer.

Mass spectra data were processed using Data Explorer 4.9 Software (Applied Biosystems). The processed spectra were subjected to manual assignment and annotation with the aid of a glycobioinformatics tool, GlycoWorkBench.^60^ The proposed assignments for the selected peaks were based on 12C isotopic composition together with knowledge of the biosynthetic pathways.

#### RNA-seq and bioinformatic analysis

We generated RNA-Seq datasets from BeWo cells treated with and without Tg (5 independent replicate pairs, single-end 50bp) (Table S13). Raw fastq data have been deposited in Array Express with accession number E-MTAB-10943 (https://www.ebi.ac.uk/arrayexpress/experiments/E-MTAB-10943/). The alignment and QC were processed using the nextflow (version 21.05.0.edge)^72^ pipeline nf-core/rnaseq (version 3.2, https://nf-co.re/rnaseq; Ewels et al., 2020)^61^ with the option “--aligner star_salmon” and Ensembl reference genome and annotation for human GRCh38.104. Tables S14 and S1 show the number of raw reads, mapped reads, and the mapping statistics. All scripts, with details of software versions, a pipeline usage report and expression raw count files are in Table S15 and freely available from https://github.com/CTR-BFX/Yung_Charnock-Jones and Zenodo, (https://doi.org/10.5281/zenodo.7373310).

There are 60504 genes identified using the nextflow pipeline in total with Ensembl Gene ID annotation. First, we filtered the low counts by limiting the total number of normalised reads counts on the estimated size factors to be greater than 10 for each gene. 23426 genes were used to perform differential gene expression analysis using DESeq2 package (version 1.30.1)^62^ in R (version 4.0.2) (https://www.R-project.org/). The design formula for DESeq2 analysis is “∼Paired + Condition”. After the DESeq2 analysis, a further 2725 genes were removed due to missing p-adjusted values. The final number of genes used for subsequent analyses was 20701. Normalised counts and corrected p values for the differentially expressed genes (DEGs) are listed in Suppl. Tables S16 and S2 respectively.

Principal component analysis (PCA) was performed using the most variable 2000 genes with variance stabilising transformed expression for gene counts (Figure S2A). The gene ontology analysis was performed using goprofiler2 package (version 0.2.1)^63^ with 1712 up and 1482 down regulated genes, respectively.

Further analyses to show the relationship between DEGs and gene ontologies of interest are presented as Heatmaps, and GeneSet enrichment (GSE) plots. Heatmaps were generated using R package ComplexHeatmap (version 2.6.2).^64^ Data sets used to perform the corresponding Heatmap Figures are given in Tables 4, S5 and S6. GSE analysis was performed using GESA software (v4.1.0) (http://www.gsea-msigdb.org/gsea/index.jsp) with gene sets database (go.bp.v7.4.symbols.gmt[Gene ontology]).^73^ There are 884 biological processes identified for thapsigargin (Table S3).

#### Reverse transcription and quantitative real-time PCR

Reverse transcription was performed as described previously.^74^ In brief, total RNA was extracted using either an RNeasy Mini Kit (Qiagen, 74104) for cells or RNeasy Plus Universal Mini Kit (Qiagen, 73404) for tissues following the manufacturer’s instructions. 1 μg of total RNA was used for cDNA synthesis using SuperScript III Reverse Transcriptase (ThermoFisher Scientific, UK) using random hexamers.

##### PCR analysis of Vegfa mRNA splice variants

The PCR primers used for *Vegfa* splice variant analysis are listed in Table S11. PCR was performed using Taq DNA polymerase (10342020, ThermoFisher Scientific, UK). PCR products were resolved in agarose gel containing ethidium bromide and documented in a UVP Gel Documentation system (UVP, UK).

##### Quantitative real-time PCR

qPCR was performed using either SYBR® Green JumpStart™ Taq ReadyMix™ (Sigma, S4438) or Absolute qPCR ROX Mix (Thermo Scientific, AB-1138/B) according to the manufacturer’s instructions using the OPTICON2 thermocycler (MJ Research, UK). Primer sequences are listed in Table S11. The transcript levels were calculated using the threshold cycle method (2^−ΔΔCT^ method) with reference to the average value of 18S and TBP or Hprt1. The results were presented as relative levels.

#### Electron microscopy

Placental tissues were processed and images taken in the Cambridge Advanced Imaging Centre. In brief, placental tissues were fixed by immersion in 2% glutaraldehyde containing 2 mM CaCl_2_ in 100 mM PIPES buffer at pH 7.4, in which H_2_O_2_ was added at final concentration of 0.3% (v/v) immediately before use. The tissues were fixed overnight at 4°C. After rinsed twice in buffer (100 mM PIPES), the tissues were subjected for post-fixation in 1% osmium ferricyanide for 1 h at room temperature, then rinsed three times with distilled water before staining in 2% uranyl acetate for 1 h and dehydrated in ascending concentrations to 100% ethanol. They were rinsed twice in acetonitrile and embedded in Quetol epoxy resin. Fifty-nanometer sections were cut on a Leica Ultracut UCT, stained with saturated uranyl acetate in 50% ethanol and lead citrate, and viewed in a FEI Philips CM100 operated at 80 kV.

#### Lectin staining

Reagents were from Fisher Scientific (Loughborough, UK) unless stated otherwise.

Placentae fixed in 4% paraformaldehyde were cut into two 1 mm-wide longitudinal strips before post-fixation in 2.5% EM grade glutaraldehyde (R1010, Agar Scientific Ltd., Stansted, UK) in 100 mM sodium cacodylate buffer pH 7.2 (S006, TAAB Laboratories equipment, Aldermaston, UK) for 4 h at room temperature and then rinsed 3 times in buffer over 24 h, processed through an ascending alcohol series and propylene oxide (P021, TAAB Laboratories Equipment) and flat embedded, cut surface down, in TAAB epoxy resin (T004, TAAB Laboratories Equipment Ltd).

Epoxy resin sections 0.75 μm thick were cut on a Reichert-Jung Ultracut ultramicrotome, mounted on (3′Aminopropyl)triethoxysilane coated slides (A3648, Sigma-Aldrich, Merck, Darmstadt, Germany) and dried at 50°C for 2 days. Resin was removed with saturated sodium ethoxide diluted 1: 1 with absolute ethanol for 12 min, followed by washes in ethanol and distilled water, then endogenous peroxidase was quenched for 8 min with 10% aqueous hydrogen peroxide before exposure to 0.03% trypsin from porcine pancreas (T7409, Type II-S; Sigma-Aldrich) in 50 mM Tris-buffered saline (TBS), pH 7.6, with added 1% CaCl_2_ for 4 min at 37°C. After washing in water, sections were incubated in 10 μm/mL biotinylated lectin (Concanavalin A, *P. sativum* and *D. stramonium* Agglutinins (B-1005, B-1055, B-1185) Vector Laboratories, Peterborough, UK) in 50 mM TBS containing 1 mM calcium chloride for 75 min at 37°C, washed 3 times 5 min in the same buffer, then treated with 5 μg/mL avidin peroxidase (A3151, Sigma-Aldrich) in 125 mM TBS, pH 7.6, with added 347 mM sodium chloride for 90 min at 37°C. Sections were washed in 50 mM TBS 3 times 5min and sites of lectin binding revealed with 0.05% (w/v) 3′3′diaminobenzidine tetrahydrochloride dihydrate (26,189-0, Sigma-Aldrich) in 50 mM TBS, pH 7.6, and 0.015% hydrogen peroxide (100 vol) for 7 min at 18°C. Sections were rinsed, air-dried and mounted in DPX mounting medium. Negative controls were carried out by substitution of 50 mM TBS (pH 7.6) with added calcium for the lectin, in order to identify any nonspecific binding of avidin peroxidase to the tissue section, or the presence of residual endogenous peroxidase activity.

#### Glycogen assay

A colorimetric glycogen Assay kit (ab169558, Abcam UK) was used to determine glycogen content in liver samples and the procedures were carried out following manufacturer’s instructions. In brief, liver lysates were prepared with a MagNA Lyser Instrument (Roche) using Lysing Matrix D (MP Biomedicals, UK). The tissue lysates were boiled at 100°C to inactivate endogenous enzymes before centrifuging for 5 min at 12000 rpm. The clarified supernatant was transferred to new tube and glycogen content determined according to the manufacturer’s instructions. BCA was used to measure protein concentration in tissue lysate and was used for normalisation.

### Quantification and statistical analysis

All statistical analyses were performed using GraphPad Prism 9. Details of statistical analysis for each study are included in the respective figure legend. All N-values in figure legends were the number of independent biological replicates. All tests were two-sided and p < 0.05 was defined as statistically significant. Datasets were tested for normality using the D'Agostino-Pearson omnibus test and Shapiro-Wilk test. Non-parametric tests were used for datasets that were not normally distributed.

## Data Availability

• Bewo RNA-seq data have been deposited at Array Express and are freely available with accession number E-MTAB-10943 (https://www.ebi.ac.uk/arrayexpress/experiments/E-MTAB-10943/). Accession numbers are also listed in the key resources table. • All original code has been deposited at Zenodo and is publicly available as of the date of publication. DOIs are listed in the key resources table. • Any additional information required to reanalyze the data reported in this paper is available from the lead contact upon request.

## Supplemental information

Document S1. Figures S1–S13

Table S1. Mapping statistics, related to Figure 2

Table S2. Significant differentially expressed gene list, related to Figure 2

Table S3. GSEA report for thapsigargin, related to Figure 2

Table S4. Gene list for GSEA_GOBP_ER_unfolded protein response, related to Figure 2

Table S5. Gene list for GSEA_GOBP_ER_Mannose_Trimming, related to Figure 2

Table S6. Gene list for GSEA_GO_BP_Protein_Deglycosylation, related to Figure 2

Table S7. Gene list for GO:0006486 FC > 1.5, padj<0.05, related to Figure 2

Table S8. Clinical characteristics for NC and ePE maternal serum samples, related to Figure 6

Table S9. Proteins identified by MS in WGA-isolated ePE and NC serum samples, related to Figure 6

Table S10. Clinical characteristics for NTC and ePE placental samples, related to Figure 6

Table S11. Primer sequences or sources used for genotyping, conventional RT-PCR and RT-qPCR, related to STAR Methods

Table S12. Antibody information, related to STAR Methods

Table S13. RNA_Seq sample table summary, related to STAR Methods

Table S14. Sample Table Input for nextflow, related to STAR Methods

Table S15. Nextflow software and corresponding versions, related to STAR Methods

Table S16. Normalized counts for each gene across all samples, related to STAR Methods
