# Supplementary material for: Perturbation of placental protein glycosylation by endoplasmic reticulum stress promotes maladaptation of maternal hepatic glucose metabolism
Source: iScience. 2022 Dec 30;26(1):105911. doi: 10.1016/j.isci.2022.105911 (PMC9843443; doi:10.1016/j.isci.2022.105911)
Supplement: Document S1. Figures S1–S13 [file mmc1.pdf]

iScience, Volume *26*

Supplemental information

Perturbation of placental protein glycosylation by endoplasmic reticulum stress promotes maladaptation of maternal hepatic glucose metabolism

Hong Wa Yung, Xiaohui Zhao, Luke Glover, Charlotte Burrin, Poh-Choo Pang, Carolyn J.P. Jones, Carolyn Gill, Kate Duhig, Matts Olovsson, Lucy C. Chappell, Stuart M. Haslam, Anne Dell, Graham J. Burton, and D. Stephen Charnock-Jones

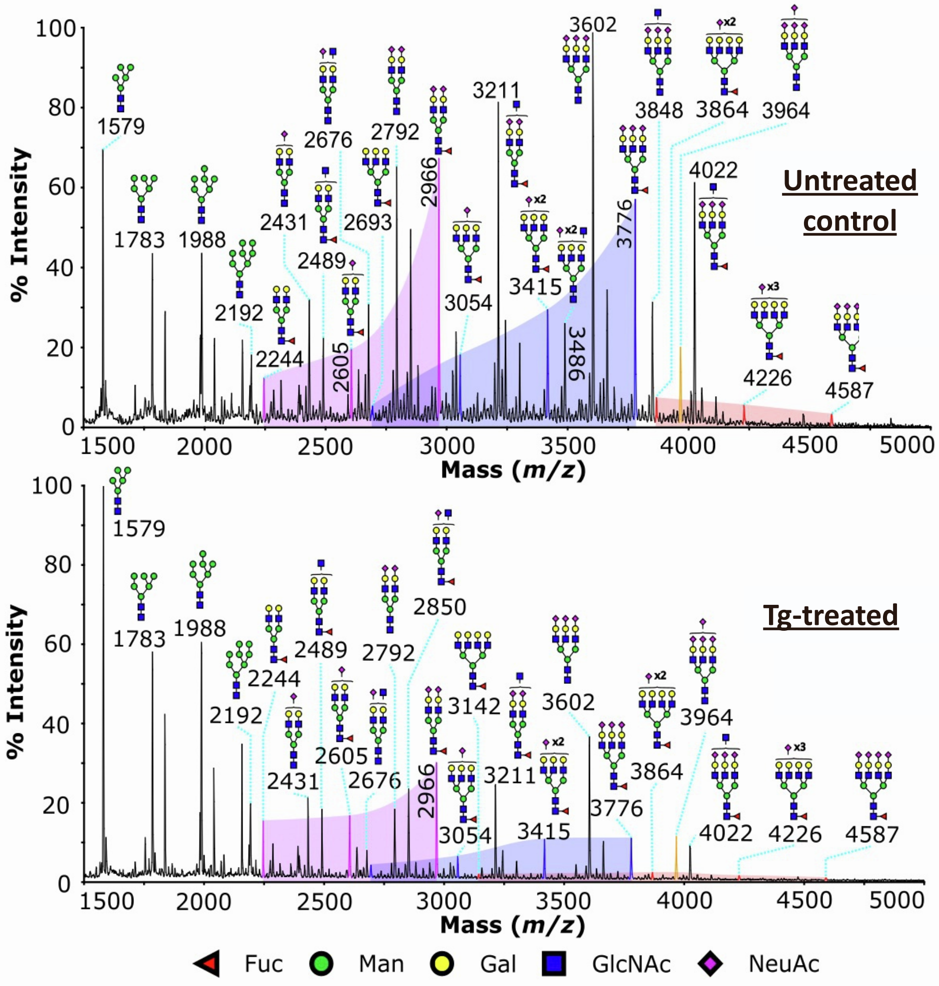

Suppl. Fig. S1: **Glycomic analysis of conditioned medium from untreated and Tg-treated BeWo cells.** BeWo cells were treated with ER stress inducers, thapsigargin (Tg) (500 nM) in serum-free medium for 24 h. The conditioned media were harvested. MALDI-TOF mass spectra of the glycans released from proteins secreted by untreated control (upper) and Tg-treated (lower) BeWo cells. Tg treatment induced two striking set of changes: i) Reduced complexity of the glycans. There is a decrease % of intensity of peaks with high mass (>2400 m/z) while an increase % of intensity of peaks with low mass (m/z 2192, 1988, 1783 and 1579) in Tg-treated CM. ii) Impaired degree of glycan sialylation. The magenta, blue and red shaded areas highlight the distribution shift of sialylation on the corresponding bi-, tri- and tetra-antennary N-glycans. The individual magenta, blue and red peaks correspond to bi-, tri- and tetra-antennary N-glycans with various levels of sialylation, respectively. Non-core fucosylated structures corresponded to residual fetal bovine serum-derived N-glycans that we also characterized previously (orange peaks; m/z 2792, 3602, and 3964). These N-glycans were observed consistently in all samples and were excluded from the analyses as previously described. Structures outside a bracket have not been unequivocally defined. Putative structures are based on composition and knowledge of biosynthetic pathways. All molecular ions are [M+Na]+. The graphs were representative of 2 independent experiments. Related to Figure 1.

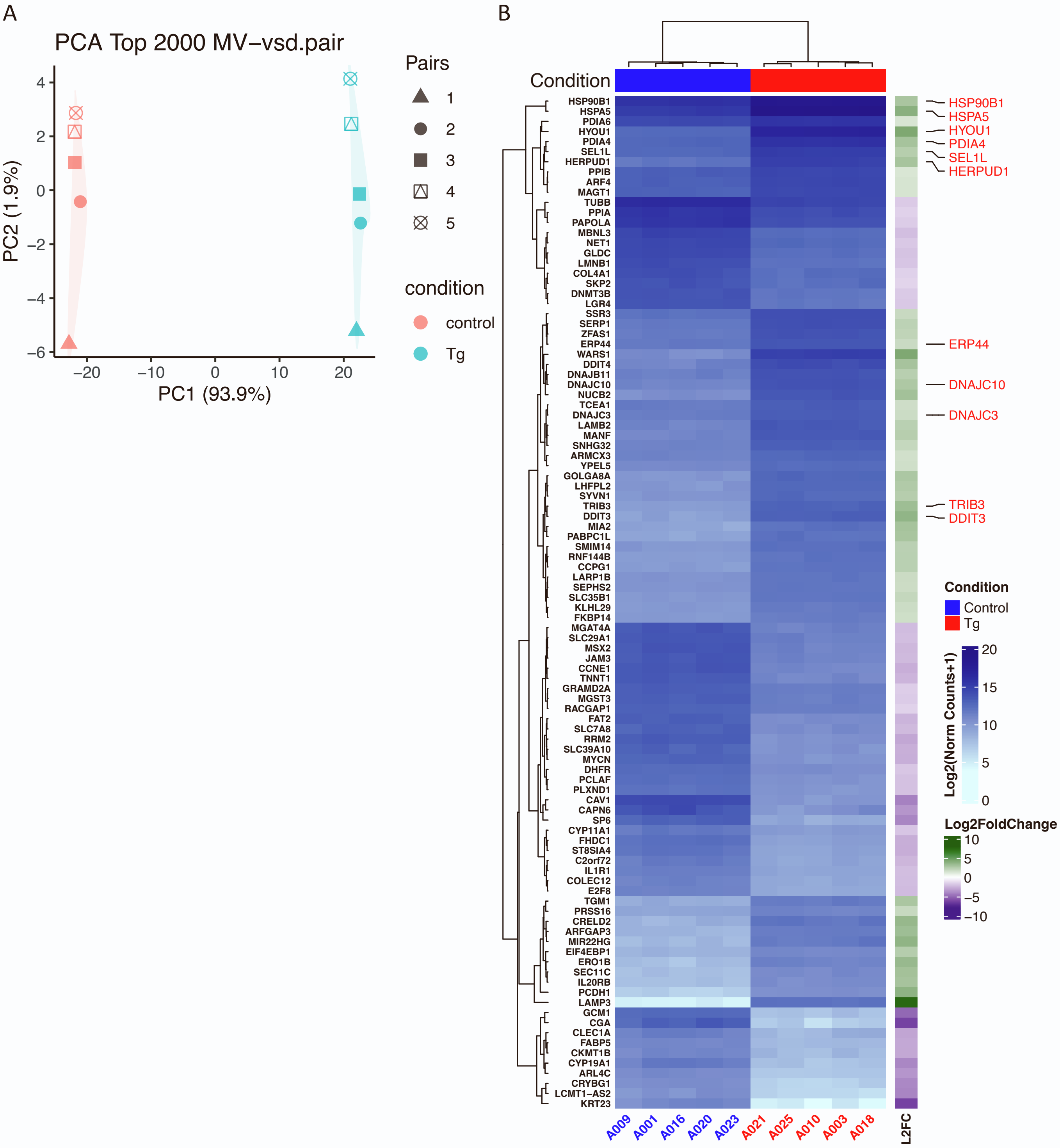

***Suppl. Fig. S2:* Genomic analysis in BeWo cells after Tg treatment. A)** Principal Component Analysis. Even though there appears to be a wide spread of samples due to variation in PC2 this only accounts for 1.9% of the variation among these samples. **B)** Clustering and heatmap analysis of top 100 DEGs. The genes involve in ER stress or ER-UPR were highlighted in red on the right. Related to Figure 2.

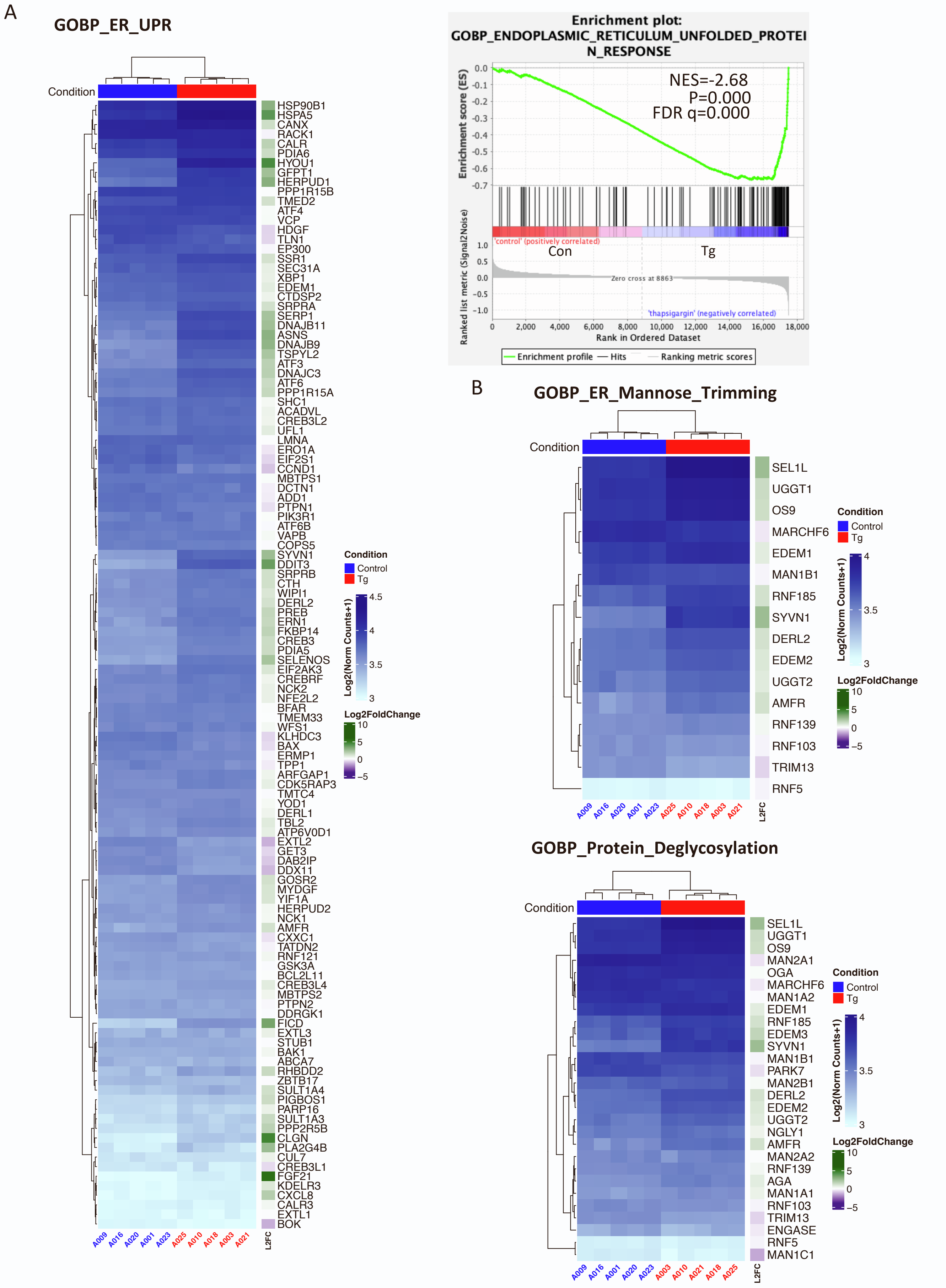

***Suppl. Fig. S3:* GSE analysis of 20701 genes from processed RNA-Seq datasets in BeWo cells after Tg treatment. A)** Enrichment plot of GOBP_Endoplasmic_Reticulum_Unfolded_Protein_Response and the corresponding heatmap of enriched genes. **B)** Heatmap of enriched genes for GOBP_ER_Mannose_Trimming and GOBP_Protein_Deglycosylation. Related to Figure 2.

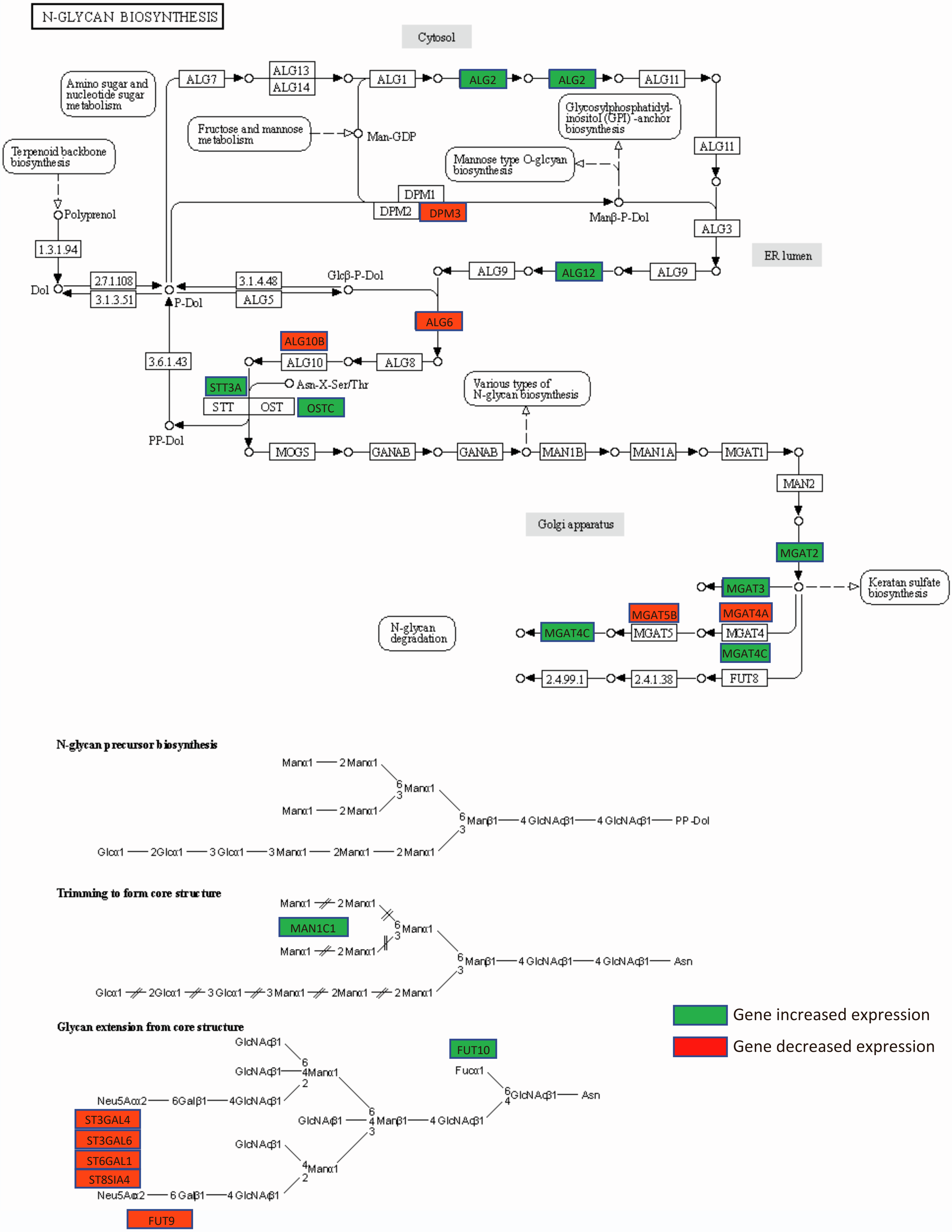

Suppl. Fig. S4: **Illustration potential mechanisms for ER stress in modulation of N-link glycosylation.** The 66 DEGs involving in protein glycosylation was incorporated into the KEGG pathway for N-glycan biosynthesis (KEGG: 00510). Related to Figure 2.

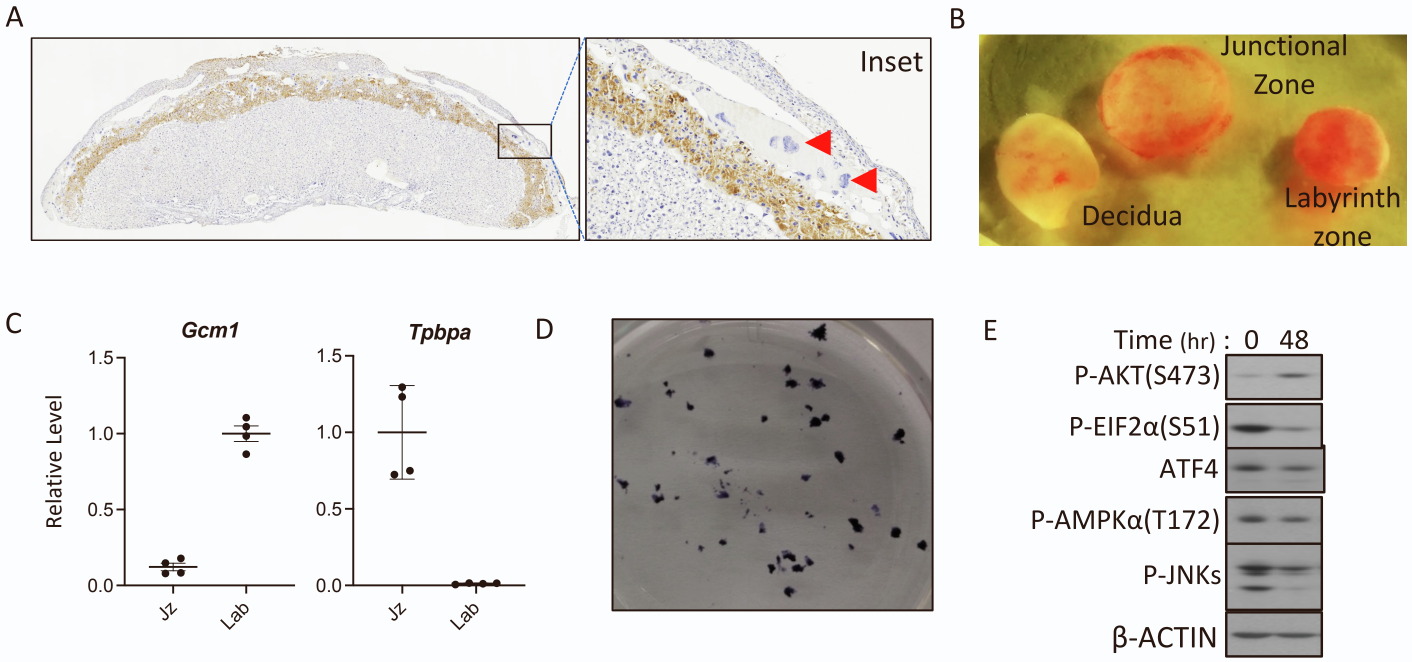

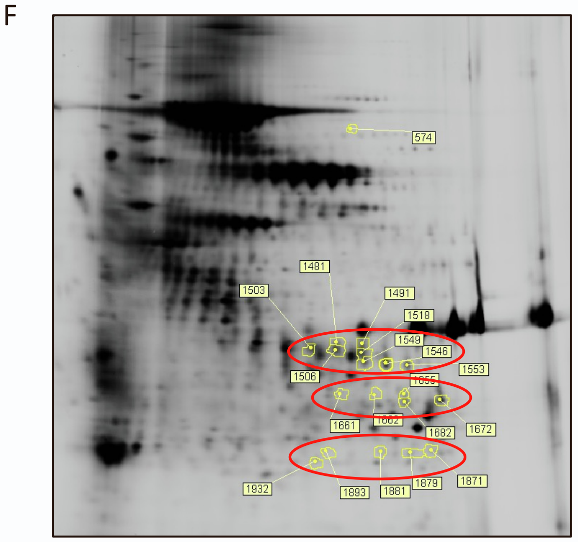

G

Spot ID	Accession Number	Description
1661	tr|Q3TS44|Q3TS44_MOUSE	Proteasome subunit alpha type OS=Mus musculus GN=Psma1 PE=1 SV=1
1503	tr|Q4KL40|Q4KL40_MOUSE	Carcinoembryonic antigen-related cell adhesion molecule 11 OS=Mus musculus GN=Ceacam11 PE=2 SV=1
1932	tr|Q497W2|Q497W2_MOUSE	Pregnancy-specific glycoprotein 27 OS=Mus musculus GN=Psg27 PE=1 SV=1
1481	-	
1871	tr|Q9Z1R9|Q9Z1R9_MOUSE	MCG124046 OS=Mus musculus GN=Prss1 PE=1 SV=1
1506	tr|Q4KL40|Q4KL40_MOUSE	Carcinoembryonic antigen-related cell adhesion molecule 11 OS=Mus musculus GN=Ceacam11 PE=2 SV=1
1879	tr|Q9Z1R9|Q9Z1R9_MOUSE	MCG124046 OS=Mus musculus GN=Prss1 PE=1 SV=1
1553	tr|Q4KL40|Q4KL40_MOUSE	Carcinoembryonic antigen-related cell adhesion molecule 11 OS=Mus musculus GN=Ceacam11 PE=2 SV=1
1881	tr|Q8BJX0|Q8BJX0_MOUSE	Proteasome subunit beta type OS=Mus musculus GN=Psmb2 PE=2 SV=1
1546	tr|Q4KL40|Q4KL40_MOUSE	Carcinoembryonic antigen-related cell adhesion molecule 11 OS=Mus musculus GN=Ceacam11 PE=2 SV=1
1655	tr|Q3UKP4|Q3UKP4_MOUSE	Protein Ceacam12 OS=Mus musculus GN=Ceacam12 PE=1 SV=1
1491	tr|Q4KL40|Q4KL40_MOUSE	Carcinoembryonic antigen-related cell adhesion molecule 11 OS=Mus musculus GN=Ceacam11 PE=2 SV=1
574	sp|Q9CWJ9|PUR9_MOUSE	Bifunctional purine biosynthesis protein PURH OS=Mus musculus GN=Atic PE=1 SV=2
1682	tr|Q3UKP4|Q3UKP4_MOUSE	Protein Ceacam12 OS=Mus musculus GN=Ceacam12 PE=1 SV=1
1518	tr|Q4KL40|Q4KL40_MOUSE	Carcinoembryonic antigen-related cell adhesion molecule 11 OS=Mus musculus GN=Ceacam11 PE=2 SV=1
897	tr|Q3TUD6|Q3TUD6_MOUSE	Putative uncharacterized protein OS=Mus musculus GN=Hsp90b1 PE=2 SV=1
1672	tr|Q3UKP4|Q3UKP4_MOUSE	Protein Ceacam12 OS=Mus musculus GN=Ceacam12 PE=1 SV=1
1549	tr|Q4KL40|Q4KL40_MOUSE	Carcinoembryonic antigen-related cell adhesion molecule 11 OS=Mus musculus GN=Ceacam11 PE=2 SV=1
1893	tr|Q8BJX0|Q8BJX0_MOUSE	Proteasome subunit beta type OS=Mus musculus GN=Psmb2 PE=2 SV=1

***Suppl. Fig. S5:* Verification of newly established junctional zone explant culture. A)** ER chaperone GRP78 immunostaining in placenta at E18.5 reveals that spongiotrophoblasts in Jz containing very high density of rough endoplasmic reticulum, consistent with its high endocrine activity. Inset, image showing that the trophoblast glycogen cells (red arrows) do not stain for GRP78. **B)** Images show placenta successfully dissected into decidual layer, junctional zone layer (Jz) and labyrinthine (Lz) zone layer. **C)** RT-qPCR confirms enriched corresponding cell types in microdissected Jz and Lz tissues. *Tpbpa* and *Gcm1* genes were used as markers for Jz and Lz respectively. Data was mean±SD, n=4. **D)** MTT staining of explants shows high viability of Jz explants after 48 h of culturing. **E)** Molecular markers were used to confirm high cell survival and proliferation-associated signalling while low cellular stress signalling in the Jz explants after 48 h culturing using western blotting analysis. **F)** Two-Dimension Fluorescence Difference Gel Electrophoresis (DIGE) shows differentially expressed proteins and the spots which were excised for analysis by LC-MS/MS. Conditioned media from untreated- and Tg-treated Jz explants were Cy3 or Cy5 labelled before 2D gel analysis. **G)** The table shows the identified proteins from through 19 spots after LC-MS/MS analysis. Related to Figure 3.

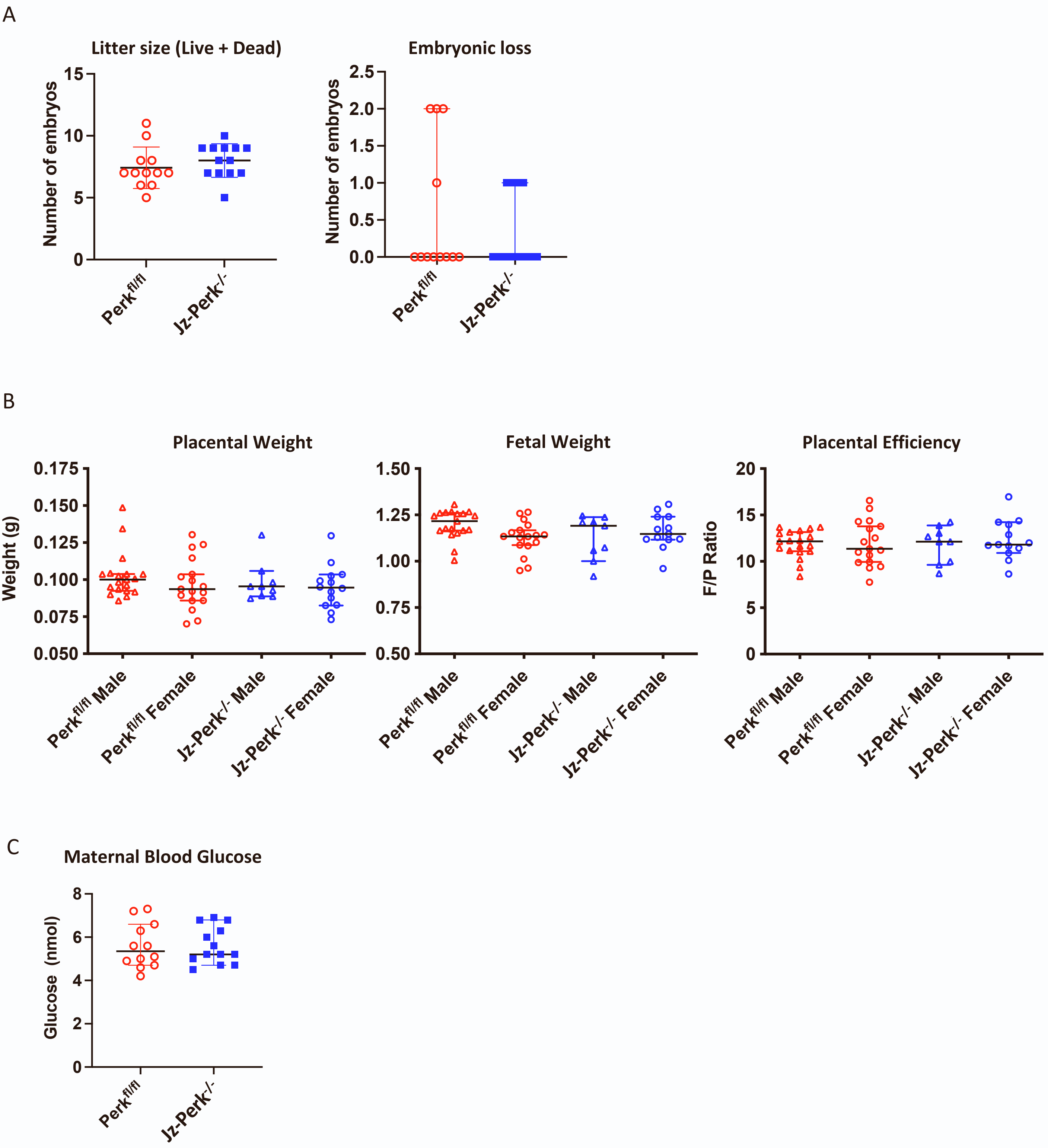

Suppl. Fig. S6: **Difference in pregnancy outcomes and maternal blood glucose in females carrying litters with** ***$Perk^{fl/fl}$*** **or** ***$Jz\text{-}Perk^{-/-}$*** **placentas housed in normal atmospheric O_2.** The females were housed under atmospheric oxygen throughout pregnancy. At E18.5, females were culled and the litter size, number of resorptions, late embryonic loss, placental and fetal weight determined. The maternal blood glucose concentration was also measured. Data is presented as mean±SD for parametric tests and is median with 95% CI for non-parametric tests. **A)** Litter size and embryonic loss. Unpaired t-test for litter size and Mann Whitney test for embryonic loss. **B)** Placental and fetal weight and placental efficiency. Kruskal-Wallis test with Dunn's multiple comparisons test. **C)** Maternal blood glucose concentration. Mann Whitney test. Related to Figure 4.

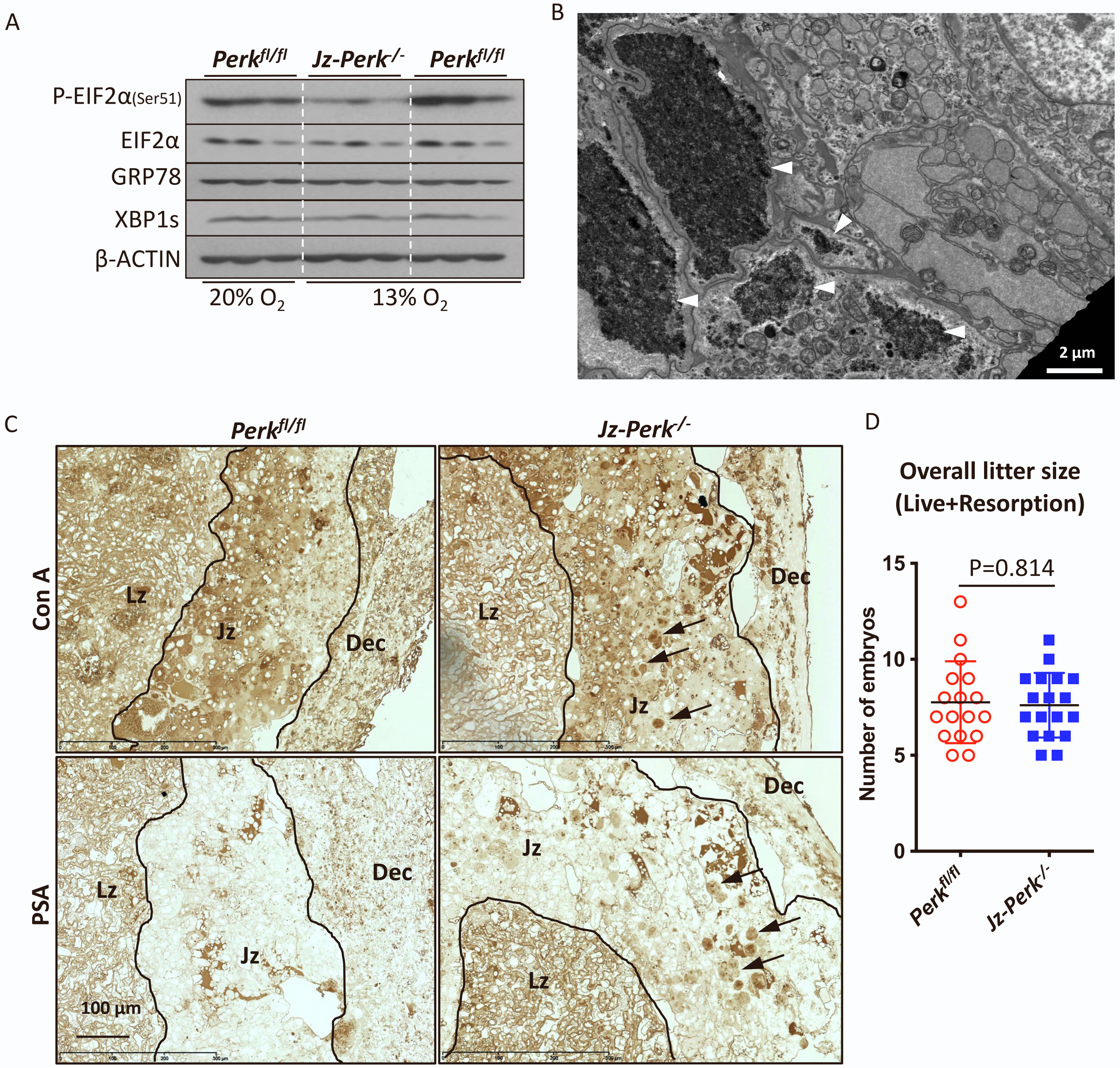

Suppl. Fig. S7: **Reduction of EIF2α phosphorylation and presence of misglycoyslated protein aggregates/deposits in junctional zone of *Jz-Perk*$^{-/-}$ placentas.** The females were housed under 13% O_2 concentration during pregnancy. At E18.5, females were culled and placentas were harvested for western blotting, electron micrography and staining of lectins. **A)** Western blot analysis of EIF2α phosphorylation status and ER stress responsive proteins in junctional zone (Jz). Dissected Jz placental tissues were used for analysis with corresponding primary antibodies. β-ACTIN was used for loading control. **B)** Electron micrograph shows various size protein deposits (arrowheads) in spongiotrophoblast of the *Jz-Perk*$^{-/-}$ placenta. **C)** Lectin staining with Concanavalin A (Con A) and Pisum sativum agglutinin (PSA). Both lectins prefer binding to α-linked mannose and reveals protein aggregates in Jz of *Jz-Perk*$^{-/-}$ placenta, indicating potential protein misglycosylation. **D)** No change in overall litter size (live and resorption) in litters with *Perk*$^{fl/fl}$ and *Jz-Perk*$^{-/-}$ placenta. Data is presented as mean±SD, *Jz-Perk*$^{-/-}$ = 16; *Perk*$^{fl/fl}$ = 16. Unpaired t-test. Related to Figure 4.

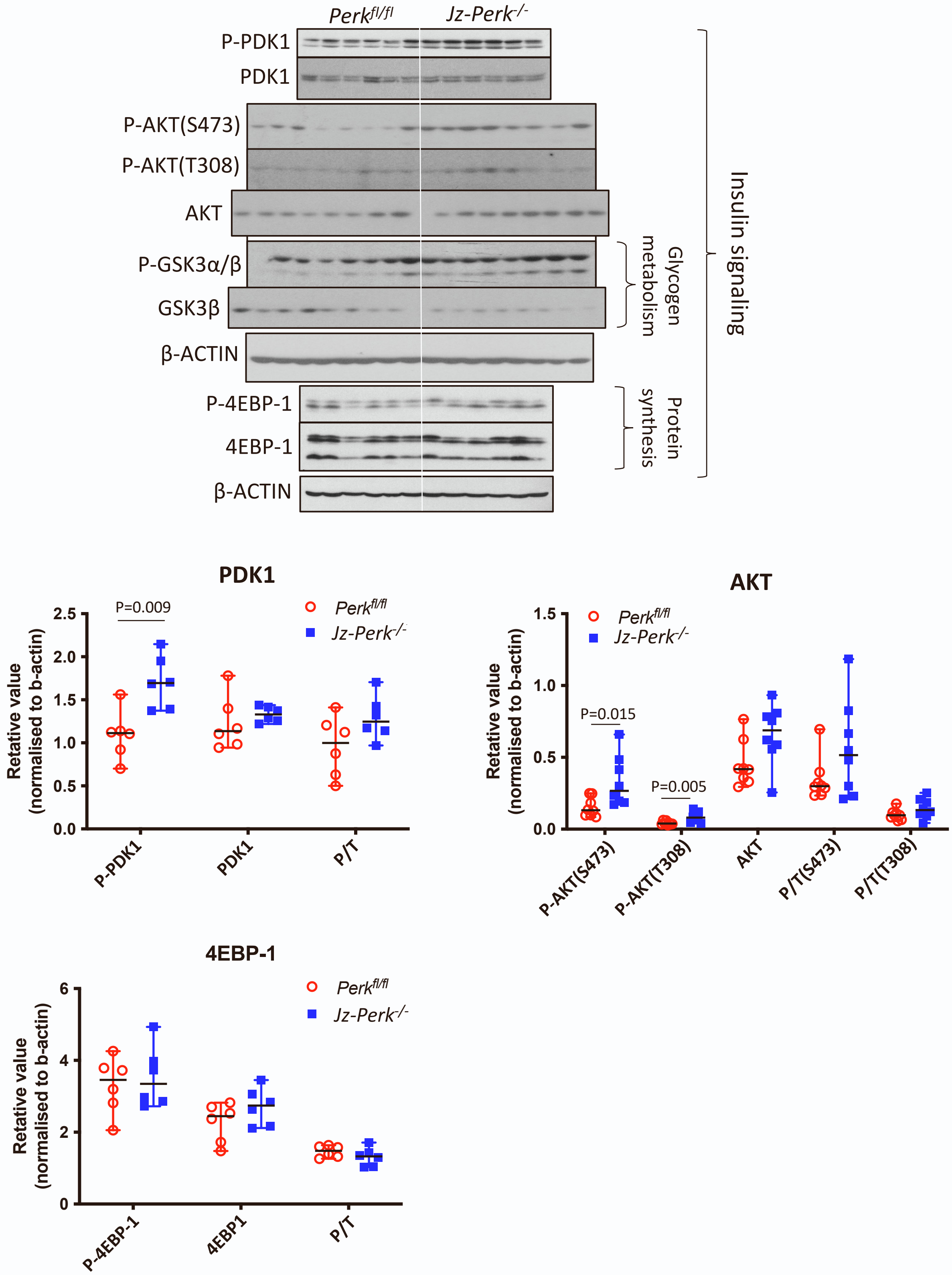

Suppl. Fig. S8: **Maternal liver in females carrying litter of *Jz-Perk$^{-/-}$* placentas increases PDK1-AKT-GSK signalling but not mTOR-4EBP1 compared to carrying litter of *Perk$^{fl/fl}$* placentas.** The females were housed under 13% O_2 concentration during pregnancy. At E18.5, females were culled and maternal liver was harvested for western blotting analysis for PDK1-AKT-mTOR signalling pathways using corresponding primary antibodies. Data is presented as median with 95% CI, n=6-8. Multiple Mann Whitney test. Related to Figure 5.

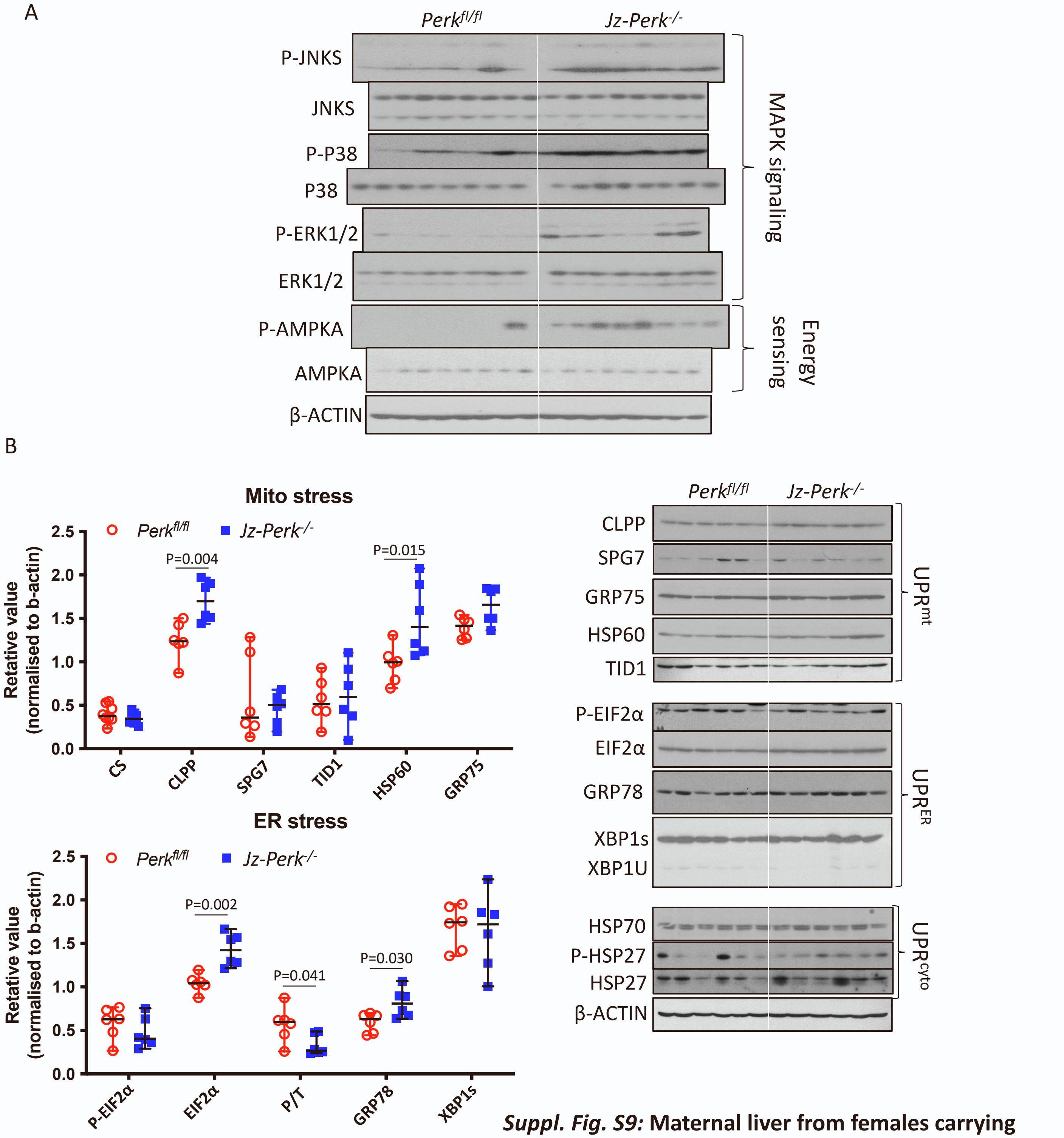

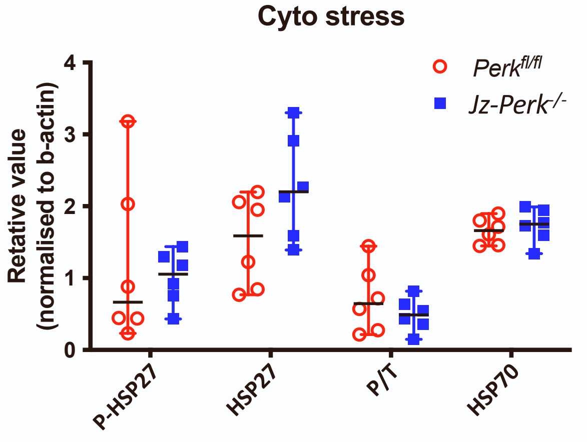

***Suppl. Fig. S9:* Maternal liver from females carrying litter of *Jz-Perk*$^{-/-}$ placentas suffers from high level of cellular stresses compared to carrying litter of *Perk*$^{fl/fl}$ placentas.** The females were housed under 13% O_2 concentration during pregnancy. At E18.5, females were culled and maternal liver was harvested for western blotting for different cellular stress signaling pathways including MAPK family, AMPKα, mitochondrial stress markers, ER stress markers, and cytosolic stress markers using their corresponding primary antibodies. Data is presented as median with 95% CI, n=6-8. Multiple Mann Whitney test. Related to Figure 5.

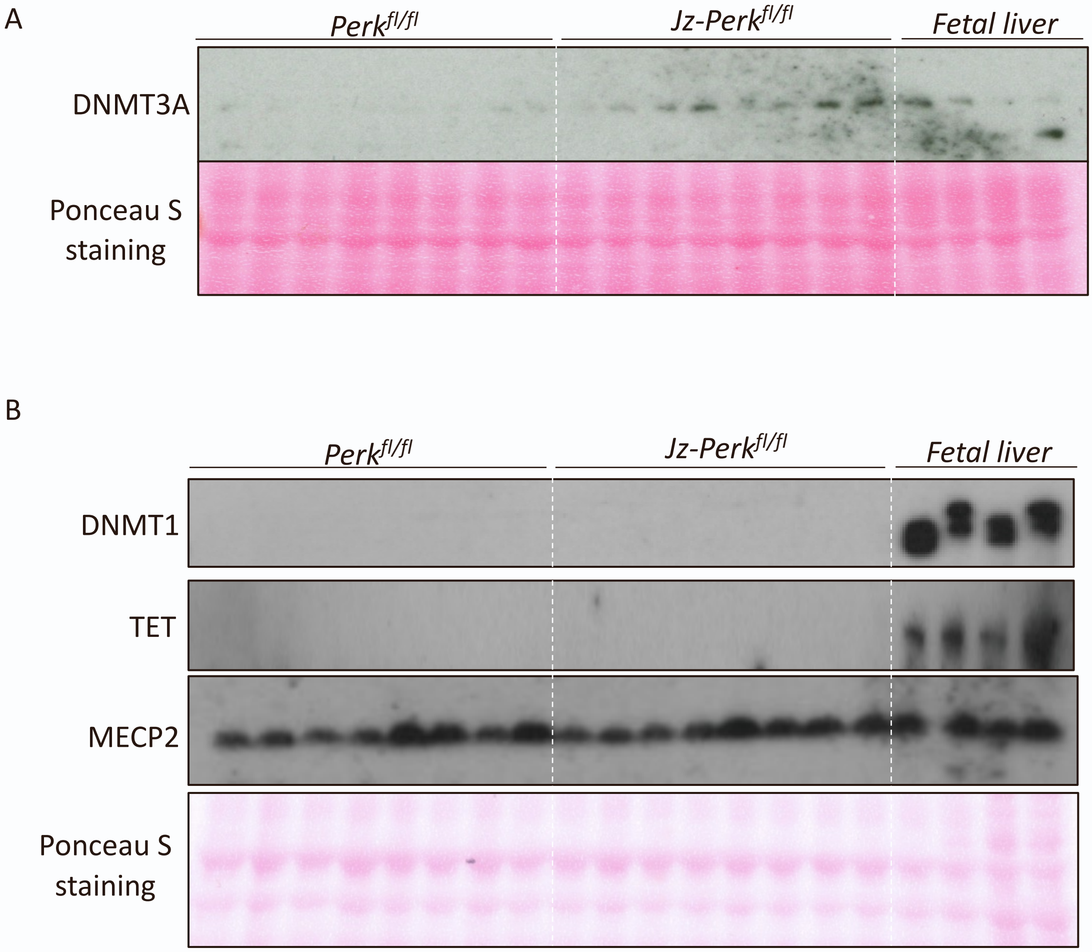

Suppl. Fig. S10: **Maternal liver from females carrying litter of *Jz-Perk*$^{-/-}$ placentas expresses high level of DNA methyltransferase, DNMT3A compared to carrying litter of *Perk*$^{fl/fl}$ placentas.** The females were housed under 13% O_2 concentration during pregnancy. At E18.5, females were culled and maternal liver was harvested for western blotting. **A)** DNMT3A. **B)** DNMT1, TET and MECP2. Fetal liver from *Perk*$^{fl/fl}$ is used as positive control. Ponceau S staining is used as loading control. Related to Figure 5.

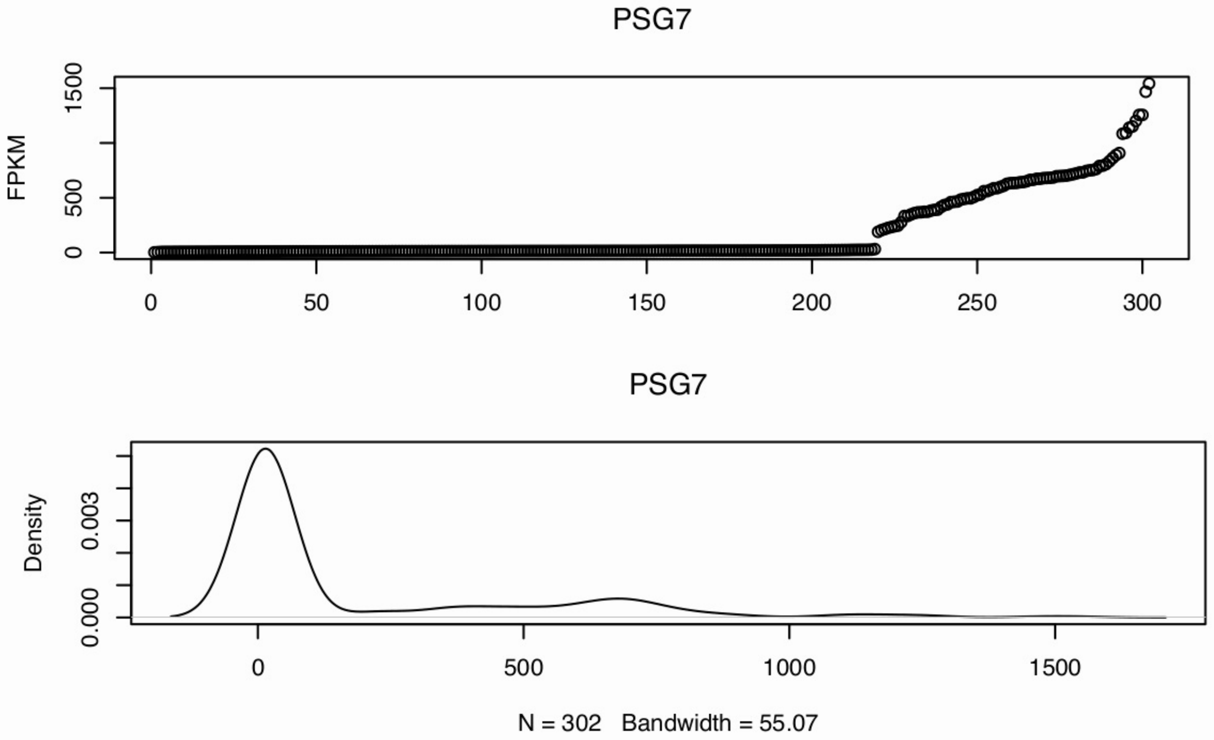

Suppl. Fig. S11: **Bimodal distribution of *PSG7* mRNA in human placenta.** RNAseq data from 302 human placentas are from Gong *et al* 2021[31]. **A)** Samples are arranged in ascending order based on *PSG7* FPKM (Fragments Per Kilobase of transcript per Million mapped reads). Two groups of sample with low and high expression levels are clearly visible. **B)** A density plot of the same data illustrating that the majority of the samples have a low level of expression while others have a considerably higher level. Related to Figure 6.

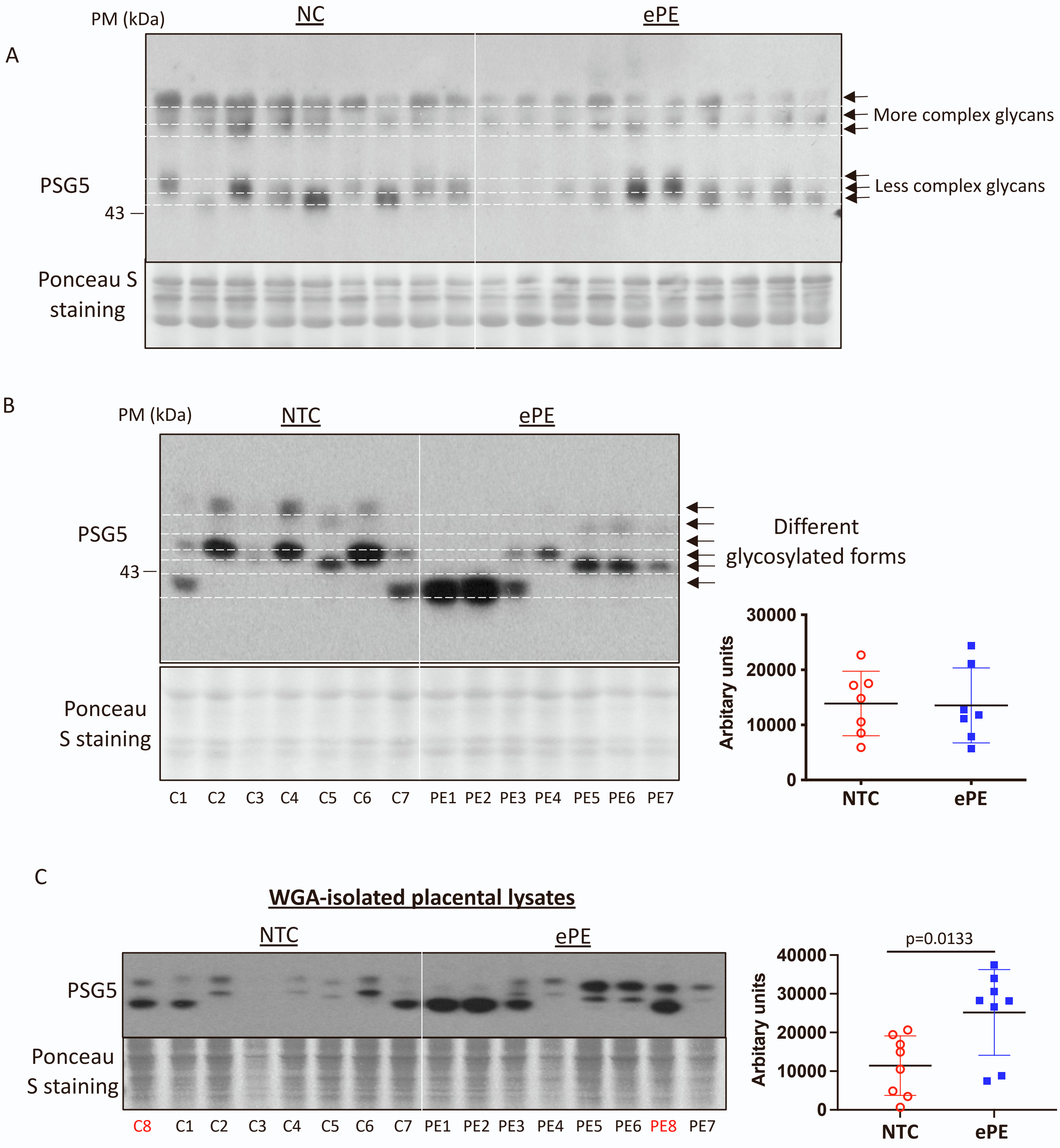

***Suppl. Fig. S12:* PSG5 shows glycosylation changes in ePE.** Western blot with prolonged gel electrophoresis was used to resolve PSG5 with different glycosylation profiles in serum or placenta. Ponceau S staining was used to show even protein loading. **A)** PSG5 glycosylation profile in normotensive control (NC) and ePE in albumin-depleted serum. The bands were grouped into "More" and "Less" complex glycans. **B & C)** PSG5 level and glycosylation profile in normotensive term control (NTC) and ePE placentas before and after WGA isolation. The dashed lines were used to aid visualisation of the glycosylation profile of PSG5. Related to Figure 6.

A

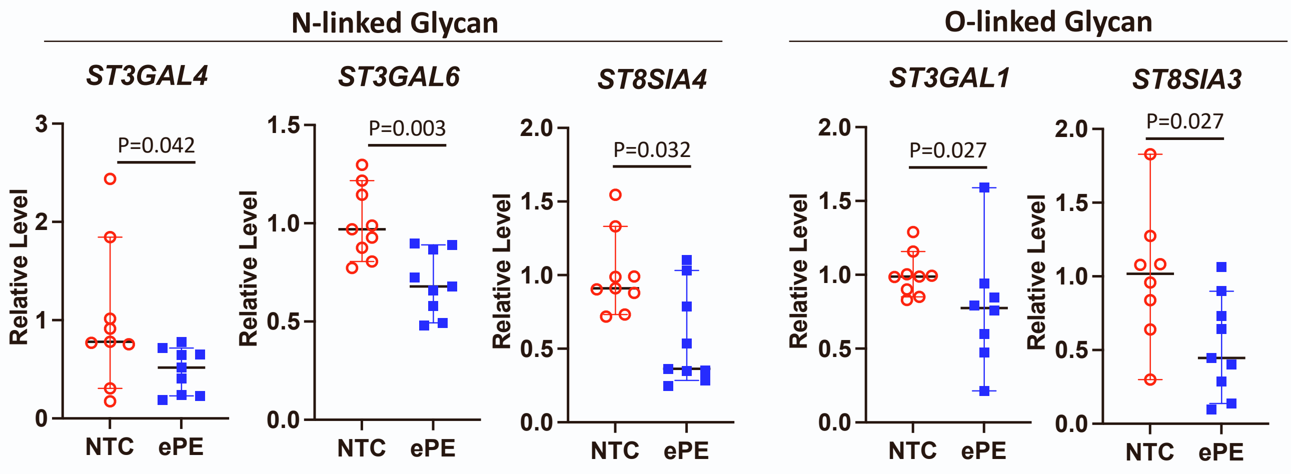

B

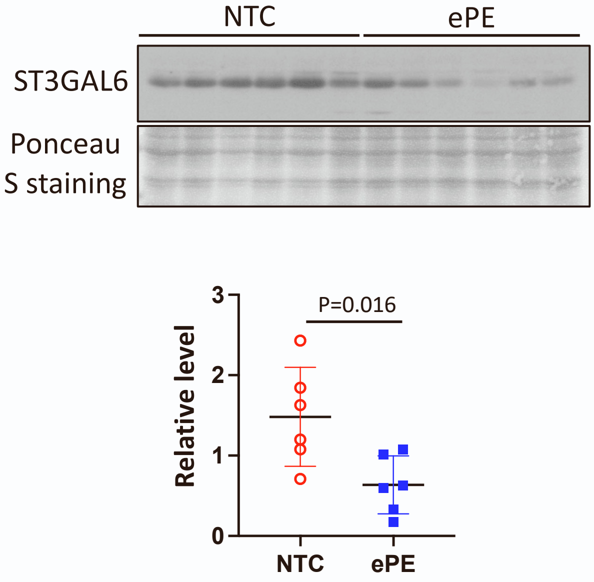

***Suppl. Fig. S13:* Sialyltransferases in N and O-linked glycosylation are reduced in the placenta in early-onset pre-eclampsia. A)** Sialyltransferase mRNAs were measured by RT-qPCR. All data was normalised to mean value of NTC before plotting and is presented as median with 95% CI, n=8. Mann Whitney U test. **B)** Reduction of ST3GAL6 level determined by Western blotting. Ponceau S staining was used normalise protein loading. Data is presented mean±SD, n=6. Unpaired t-test. Related to Figure 6.
